# Target Hopping from Protein Kinases to PXR: Identification of Small-Molecule Protein Kinase Inhibitors as Selective Modulators of Pregnane X Receptor from TüKIC Library

**DOI:** 10.3390/cells11081299

**Published:** 2022-04-12

**Authors:** Enni-Kaisa Mustonen, Tatu Pantsar, Azam Rashidian, Juliander Reiner, Matthias Schwab, Stefan Laufer, Oliver Burk

**Affiliations:** 1 Dr. Margarete Fischer-Bosch-Institute of Clinical Pharmacology, Stuttgart, and University of Tuebingen, 72074 Tuebingen, Germany; ennikaisamustonen@gmail.com (E.-K.M.); matthias.schwab@ikp-stuttgart.de (M.S.); 2 Department of Pharmaceutical and Medicinal Chemistry, Institute of Pharmaceutical Sciences, University of Tuebingen, 72076 Tuebingen, Germany; tatu.pantsar@uef.fi (T.P.); juliander.reiner@uni-tuebingen.de (J.R.); stefan.laufer@uni-tuebingen.de (S.L.); 3 School of Pharmacy, Faculty of Health Sciences, University of Eastern Finland, 70210 Kuopio, Finland; 4 Department of Internal Medicine VIII, University Hospital Tuebingen, 72076 Tuebingen, Germany; azam.rashidian@uni-tuebingen.de; 5 Departments of Clinical Pharmacology and Biochemistry and Pharmacy, University of Tuebingen, 72076 Tuebingen, Germany; 6 Cluster of Excellence iFIT (EXC 2180) “Image-Guided and Functionally Instructed Tumor Therapies”, University of Tuebingen, 72076 Tuebingen, Germany; 7 Tuebingen Center for Academic Drug Discovery & Development (TüCAD2), 72076 Tuebingen, Germany

**Keywords:** pregnane X receptor, protein kinase inhibitor, PXR antagonist, cancer therapy

## Abstract

Small-molecule protein kinase inhibitors are used for the treatment of cancer, but off-target effects hinder their clinical use. Especially off-target activation of the pregnane X receptor (PXR) has to be considered, as it not only governs drug metabolism and elimination, but also can promote tumor growth and cancer drug resistance. Consequently, PXR antagonism has been proposed for improving cancer drug therapy. Here we aimed to identify small-molecule kinase inhibitors of the Tübingen Kinase Inhibitor Collection (TüKIC) compound library that would act also as PXR antagonists. By a combination of in silico screen and confirmatory cellular reporter gene assays, we identified four novel PXR antagonists and a structurally related agonist with a common phenylaminobenzosuberone scaffold. Further characterization using biochemical ligand binding and cellular protein interaction assays classified the novel compounds as mixed competitive/noncompetitive, passive antagonists, which bind PXR directly and disrupt its interaction with coregulatory proteins. Expression analysis of prototypical PXR target genes ABCB1 and CYP3A4 in LS174T colorectal cancer cells and HepaRG hepatocytes revealed novel antagonists as selective receptor modulators, which showed gene- and tissue-specific effects. These results demonstrate the possibility of dual PXR and protein kinase inhibitors, which might represent added value in cancer therapy.

## 1. Introduction

Signaling cascades of protein kinases regulate multiple functions in cells, such as cell proliferation and survival. Their dysregulation frequently results in loss of apoptotic cell death and uncontrolled cell growth, which are among the hallmarks of cancer [1]. Thus, inhibition of protein kinases has proven to be an effective therapeutic strategy in cancer treatment; consequently, small-molecule kinase inhibitors have become a clinically important group of molecularly targeted anticancer drugs [1]. At present, approximately 70 small-molecule kinase inhibitors are approved for clinical use by the United States Food and Drug Administration (FDA) [2]. The large majority of these target tyrosine kinases, such as the anaplastic lymphoma kinase (ALK), epidermal growth factor receptor (EGFR), and vascular endothelial growth factor receptor (VEGFR). Only few inhibitors target serine/threonine kinases, such as B-RAF, or dual specificity kinases such as MEK1/2 [3].

Even if small-molecule kinase inhibitors have been conceived as molecularly targeted drugs, it is now increasingly recognized that they demonstrate pronounced off-target effects, which not only account for adverse drug effects but also may contribute to the desired activity [4]. A special case of off-target effects by protein kinase inhibitors is the modulation of the activity of xenosensing receptors of the chemical defense system, such as aryl hydrocarbon receptor or pregnane X receptor (PXR, NR1I2), a ligand-activated transcription factor of the nuclear receptor family. Activation of PXR induces drug detoxification and/or elimination, which may alter pharmacokinetics of the respective kinase inhibitors and potentially result in loss of efficacy [5]. However, PXR is not only a master regulator of drug detoxification [6], but also it was shown to modulate context-dependent tumor growth [7] and to promote cancer drug resistance [8,9], if activated in cancer cells. Therefore, PXR antagonism is proposed as a potential approach to prevent the formation of cancer drug resistance or even to overcome it [10,11]; although the mechanisms of resistance, which are caused by PXR activation, are still debated [9]. However, this approach is hindered by the limited number of specific and potent PXR antagonists and the challenging design of novel antagonists due to the promiscuous nature of PXR ligand binding [12]. Several protein kinase inhibitors activate PXR, including erlotinib, gefitinib, and sorafenib [13] or are even identified as PXR agonists, such as the MEK1/2 inhibitor U0126 [14] or dabrafenib [15]. So far only pazopanib is disclosed to elicit PXR antagonism [16]. Drugs with the dual function of inhibiting both PXR and protein kinases could open up new avenues for the treatment of cancer and in overcoming cancer drug resistance.

The objective of this study was to identify novel PXR antagonists among kinase inhibitors. To this end, using an in silico approach we screened the in-house Tübingen Kinase Inhibitor Collection (TüKIC) compound library, which contains 8500 small-molecule kinase inhibitors, to identify PXR binding compounds. Candidate ligands were subjected to experimental confirmation by PXR-dependent reporter gene assays in agonist and antagonist modes. Overall, this screening strategy resulted in the identification of four novel inhibitors and one strong activator with a common phenylaminobenzosuberone scaffold. Comprehensive characterization using different biochemical assays, assessing direct ligand binding, and cellular assays, addressing the interaction of PXR with coregulatory proteins, as well as gene expression analyses in colorectal cancer cells and differentiated hepatocytes, identified the novel inhibitors as passive mixed competitive/noncompetitive antagonists of PXR, which elicit gene- and tissue-specific modulation of PXR target gene expression.

## 2. Materials and Methods

### 2.1. Chemicals and Reagents

DMSO and 1α,25-dihydroxy vitamin D_3_ were purchased from Sigma-Aldrich (Taufkirchen, Germany). CITCO was provided by ENZO Life Sciences (Lörrach, Germany). Rifampicin was purchased from Merck Chemicals (Darmstadt, Germany). SR12813 and T0901317 were obtained from Tocris Bioscience (Bristol, UK). Minimum essential medium (MEM), Dulbecco‘s modified Eagle‘s medium (DMEM), William’s E medium, and Trypsin-EDTA solution were purchased from Thermo Fischer Scientific (Waltham, MA, USA). L-glutamine, nonessential amino acids, sodium pyruvate, and penicillin–streptomycin mixture were provided by Biozym (Hessisch Oldendorf, Germany). Fetal bovine serum (FBS) was obtained from Biowest (Nuaillé, France). Oligonucleotide primers were provided by Biomers (Ulm, Germany). TaqMan probes were purchased from Applied Biosystems (Waltham, MA, USA).

### 2.2. Origin of Compounds

**Scheme 1:**
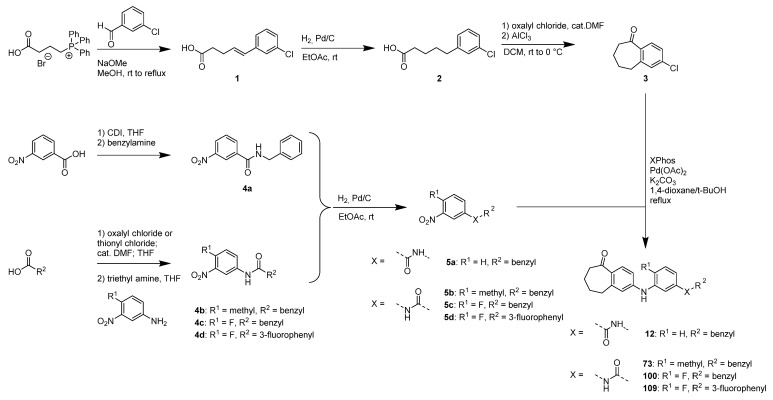
Synthesis of the described compounds; CDI = carbonyldiimidazole; DCM = dichloromethane; t-BuOH = tert-butanol.

The hit compounds of the TüKIC compound library were synthesized in-house. Synthesis of compound **2** has been reported earlier [17]. The compounds **12**, **73**, **100,** and **109** were synthesized in a convergent synthesis approach, adopted from Martz et al. [17] (Scheme 1). Further details are provided in the Supplementary Information. In brief, the benzosuberone moiety was afforded in three steps starting from 3-chlorobenzaldehyde. The latter one was brought to reaction with the previously in situ formed **4** (triphenylphosphoranylidene)butanoate in a Wittig reaction, and the double bond in the resulting unsaturated carboxylic acid **1** was subsequently reduced by catalytic hydrogenation to achieve **2**. The resulting saturated carboxylic acid was activated as carboxylic acid chloride, and the ring closure to **3** was accomplished by intramolecular Friedel–-Crafts acylation. In the synthesis of the side chains only the first step differs, depending on the nature of the amide (anilide or benzamide) and the availability of carboxylic acid chlorides. Due to the relatively higher nucleophilicity of benzylamine compared to anilines, the inverse amide **4a** could be afforded by activating 3-nitrobenzoeic acid with carbonyl diimidazole and ensuing addition of benzylamine. For the synthesis of the anilides **4b**–**d,** the carboxylic acids were activated in situ as carboxylic acid chlorides with thionyl or oxalyl chloride, and then the respective anilines were added together with triethylamine as auxiliary base. The next step was the reduction of the aromatic nitro group to aromatic amines **5a**–**d**; this succeeded for all structures in the same way as catalytic hydrogenation under palladium/charcoal catalysis. In the final step, the above-described chlorobenzosuberon scaffold and the side chains were coupled to afford the final compounds **12**, **73**, **100,** and **109** in the sense of a Buchwald–Hartwig amination reaction.

### 2.3. Molecular Modeling

All the modeling was conducted by using OPLS3e force field [18] with Maestro (2018-4; Schrödinger, LLC, New York, NY, USA). The TüKIC library of 8475 compounds was first filtered to keep only compounds with heavy atoms between 15–50, and compounds with long aliphatic chains (>6 carbons in a row) were excluded. The remaining compounds were prepared using LigPrep (Schrödinger, LLC, New York, NY, USA) with generating possible ionization states at pH 7.0 ± 2.0 using Epik [19], tautomers, and stereoisomers. Finally, compounds with a molecular weight > 600 and AlogP > 7.5 or <0 were excluded, resulting in 14,804 structures that were docked. Virtual screening was conducted by utilizing a PXR structure (PDB ID: 4j5w [20]), which prior to docking was refined by molecular dynamic (MD) simulations in complex with the competitive PXR antagonist SPA70 (data not shown), where three water molecules were kept on the site. Compounds were docked initially with Glide using extra precision (XP) accuracy [21,22,23]. The top scoring 150 compounds were redocked, using Induced Fit docking [24,25,26]; the results were visually analyzed, and selected poses underwent a further analysis by short molecular dynamics (MD) simulations, finally resulting in the selection of 56 compounds for experimental confirmation. The analogue searches at the subsequent stages were conducted by 2D structural search from the TÜKIC library.

We used the QM Conformer & Tautomer Predictor tool of Maestro (2021-2; Schrödinger LLC, New York, NY, USA) to generate five conformers for each of the five active compounds with the solvent set as water. This tool uses a stepwise minimization, applying Jaguar [27] in the final stages in the conformation generation.

### 2.4. Plasmid Constructs

Expression plasmids encoding human nuclear receptors CAR1 [28], PXR [29], RXRα [30], VDR [31], LBP-filled triple mutant PXR(S208W/S247W/C284W) [16], and CAR3 [32] have all been described previously. Expression plasmids encoding fusion proteins of GAL4-DNA binding domain (DBD) and the receptor interaction domains (RID) of steroid receptor coactivator-1 (SRC-1, residues 583–783) [32] and of silencing mediator of retinoid and thyroid hormone receptors (SMRT, residues 1109–1330) as well as the expression plasmid encoding the fusion of GAL4-DBD with PXR LBD helix 1 part (residues 132–188) [31] have been described previously. The expression plasmids encoding fusion proteins of the VP16 activation domain (AD) and PXR LBD (residues 108–434) or helix 2–12 part of it (residues 189–434) have been described previously [31]. The following firefly luciferase reporter gene plasmids have been described previously: CYP3A4 enhancer/promoter reporter gene plasmid pGL4-CYP3A4(7830Δ7208-364) [16] and pGL3(DR3)_3_Tk, with a trimer of CYP3A23 direct repeat (DR) 3 motif [33]. The pGL4-G5 luciferase reporter gene plasmid was constructed by cloning the 200 bp KpnI/HindIII insert of pGL3-G5 [32], containing the GAL4 binding site pentamer and E1b promoter into pGL4.10(luc2) (Promega, Madison, WI, USA). Renilla luciferase expression plasmid pGL4.75[hRLuc/CMV] (Promega) and Metridia luciferase expression plasmid pMetLuc2control (Takara-Clontech, Mountain View, CA, USA), both under control of the CMV promoter, were used.

### 2.5. Cell Culture

HepG2 cells (HB-8065, lot number 58341723, ATCC, Manassas, VA) and H-P cells, representing stably transfected HepG2 cells overexpressing PXR [34], were cultivated at 37 °C, 5% CO_2_ in MEM, which was supplemented with 10% FBS, 2 mM glutamine, 100 U/mL penicillin, and 100 µg/mL streptomycin. HepG2 cells were obtained at passage 74, propagated, and used in the experiments between passages 93 and 110. H-P cells were used up to passage 30 after validation of the clone.

LS174T cells (CL-188, ATCC) were cultivated at 37 °C, 5% CO_2_ in DMEM supplemented with 10% FBS, 2 mM L-glutamine, 100 U/mL penicillin, 100 µg/mL streptomycin, 1% nonessential amino acids, and 1 mM sodium pyruvate. LS174T cells were obtained at passage 104, which was then reset as 1 and used in the experiments between passages 9 and 12. In chemical treatments, phenol red-free DMEM was used, and regular FBS was replaced by dextran-coated charcoal-treated FBS. For gene expression analyses, 0.3 × 10^6^ LS174T cells were seeded per well of a 12-well plate. The next day, chemical treatment was initiated for 48–72 h, with daily medium change.

HepaRG cells (Biopredic, Rennes, France) were cultivated in phenol red-free William’s E medium, supplemented with 10% FBS, 2 mM glutamine, 100 U/mL penicillin, 100 µg/mL streptomycin, 5 µg/mL insulin, and 50 µM hydrocortisone. For gene expression analyses, 1.0 × 10^5^ cells were seeded per well of a 12-well plate. After reaching confluence, growth medium was supplemented with 2% DMSO, and cells were further cultivated for 2 weeks for differentiation into hepatocytes, as described previously [35]. Afterward, cells were adapted to induction medium (growth medium with FBS reduced to 2% and 0.2% DMSO) for 48 h. Then chemical treatment was started for another 48 h, with daily medium change.

Cells were routinely checked for contamination with mycoplasma by PCR (VenorGeM Classic, Minerva Biolabs, Berlin, Germany).

### 2.6. Cell Viability

HepG2 cells were seeded at density of 40,000 cells in 100 µL per well in white, clear bottom 96-well plates (#655098, Greiner Bio-One, Frickenhausen, Germany). On the following day, cells were treated with 3, 10, or 30 µM of test compounds for 24 h. Cell viabilities were determined by quantifying ATP content with CellTiter-Glo Luminescent Cell Viability Assay (Promega), according to the manufacturer’s instructions. Luminescence was measured using EnSpire 2300 multimode plate reader (PerkinElmer, Rodgau, Germany) for 0.1 s. After subtracting background, relative cell viability was calculated in percent by dividing the value of treated cells by the value of DMSO-treated controls.

### 2.7. Transient Transfections, Mammalian Two Hybrid, and Reporter Gene Assays

Transient batch transfection of either HepG2 or H-P cells was conducted using JetPEI transfection reagent (Polyplus, Illkirch, France), essentially as recommended by the manufacturer. To investigate PXR activation, per well of a 96-well plate, a plasmid DNA mixture of 0.3 µg pGL4-CYP3A4(-7830Δ7208-364) and 0.01 µg pGL4.75[hRluc/CMV] was diluted with 150 mM NaCl to a final volume of 25 µL. Similarly, 0.6 µL JetPEI reagent was diluted with 150 mM NaCl to 25 µL. The diluted jetPEI was added to the diluted DNA mixture and incubated at room temperature for 15 min. In parallel, H-P cells were trypsinized and counted, and the cell number was adjusted to 40,000 cells in 200 µL, per well. The transfection mixture was added to the cell suspension and pipetted into a 96-well plate (#83.3924.300, Sarstedt, Nümbrecht, Germany). After overnight incubation, cells were treated with chemicals for 24 h before cell lysis with 50 µL of passive lysis buffer (Promega). Firefly and Renilla luciferase activities were measured from 10 µL of sample with 150 µL of firefly luciferase assay solution [29] and 100 µL Renilla luciferase assay solution [36], respectively, using EnSpire 2300. Results were normalized by dividing Firefly luciferase activity by Renilla luciferase activity measured from the same well.

For testing nuclear receptor selectivity and LBP-filled PXR mutant activation, transient batch transfection was conducted as above, but with HepG2 cells and the following plasmids, with amounts per well of 0.23–0.27 µg pGL4-CYP3A4(-7830Δ7208-364) or pGL3(DR3)_3_Tk (as reporter for VDR), 0.01 µg pMetLuc2control, and 0.03 µg either CAR1, CAR3, VDR, or PXR(S208W/S247W/C284W) expression plasmids. In addition, 0.03 µg RXRα expression plasmid was added to CAR3 transfections. Metridia luciferase activity was measured from 10 µL of medium supernatant after adding 100 µL Renilla luciferase assay solution.

In mammalian two-hybrid corepressor/coactivator interaction assays, per well 0.24 µg pGL4-G5, 0.01 µg pGL4.75[hRluc/CMV], 0.03 µg expression plasmid encoding VP16-AD/PXR LBD fusion, and 0.03 µg expression plasmids encoding GAL4-DBD/SRC1-RID or GAL4-DBD/SMRT-RID fusions, respectively, were used. For the SMRT corepressor interaction assay, additionally 0.015 µg of RXRα expression plasmid was included.

For the mammalian two-hybrid PXR ligand-binding domain assembly assay, 0.24 µg pGL4-G5, 0.01 µg pMetLuc2control, and 0.03 µg each of expression plasmids encoding GAL4-DBD/PXR(132–188) and VP16-AD/PXR(189–434) fusion proteins were used per well. Otherwise, batch transfection was performed as above with HepG2 cells.

### 2.8. Limited Proteolytic Digestion

The limited proteolytic digestion assay was performed as described previously [37]. A total of 2.5% DMSO, 30 µM of T0901317, and 100 and 250 µM of test compounds were used.

### 2.9. Competitive Radioligand Binding Assay

The assay was performed by Eurofins Cerep (Celle-Lévescault, France). Compounds **100** and **109** were tested with 7 different concentrations, ranging from 0.01 µM to 10 µM, for IC_50_ determination. Compound binding was calculated as % inhibition of the binding of the radioactively labeled PXR ligand [3H]SR12813 to recombinant human PXR, as described previously [38].

### 2.10. Quantitative Real-Time PCR Analysis

Total RNA was prepared from LS174T and HepaRG cells using the NucleoSpin RNA kit (Macherey-Nagel, Düren, Germany). RNA integrity was analyzed by formaldehyde-agarose gel electrophoresis. First strand cDNA was synthesized as described earlier [37]. Relative quantification analyses were conducted with TaqMan RT-PCR utilizing the BioMark HD system and FLEX Six Gene Expression Integrated Fluidic Circuits (Fluidigm, South San Francisco, CA, USA) as described previously [34]. TaqMan gene expression assays were either the commercially available predesigned assays Hs00184500_m1 (ABCB1) and Hs00604506_m1 (CYP3A4) (Thermo Fischer Scientific) or have been described previously: CYP2B6 [31] and 18S rRNA [39]. Data were analyzed as described previously [37]. Gene expression levels were normalized to respective 18S rRNA levels.

### 2.11. Kinase Inhibition Profiling

Compounds **100** and **109** were profiled for inhibition of kinase activity at concentrations of 1 µM and 10 µM against a panel of 335 wild-type protein kinases with single measurements. The analysis was performed by ProQinase (Freiburg, Germany).

### 2.12. Statistical Analysis

In quantitative real-time PCR analysis, if the standard deviation of Ct values in technical triplicates exceeded 0.2, the outlier was omitted. In the cell viability experiments, if the coefficient of variation of technical triplicates exceeded 20%, the outlier was omitted. Data are presented as mean ± SD of at least three independent experiments, each performed in technical triplicates, if not specified otherwise in the respective figure legend. In bar charts, individual experiments are illustrated as dots. In scatter plots, mean and individual samples are shown. Statistical significances were determined using unpaired or paired *t*-test between two groups. Multiple comparisons were performed using ordinary or repeated measures one-way analysis of variance (ANOVA) with Dunnett’s multiple comparisons test. Comparisons to a hypothetical value were conducted with one sample *t*-test corrected by the method of Bonferroni. Statistical analyses were performed with GraphPad Prism 9.3.1 (San Diego, CA, USA).

## 3. Results

### 3.1. Identification of Novel PXR Inhibitors with Phenylaminobenzosuberone Scaffold

**Figure 1:**
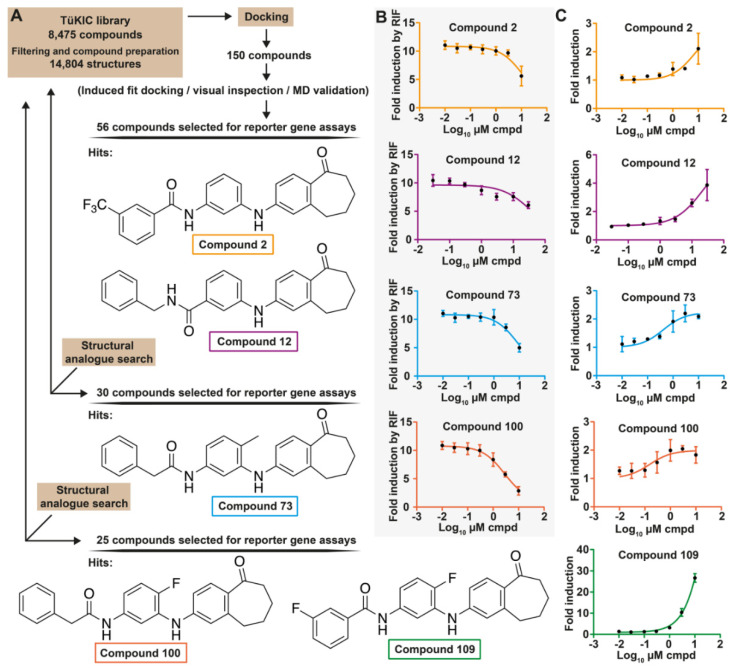
Identification of novel putative PXR ligands from the TüKIC library. (**A**) Workflow of the in silico screening and structures of the identified candidate ligands. (**B**) Concentration–response curves of the novel inhibitors for antagonism of PXR activity. (**C**) Concentration–response curves of the same compounds and **109** for PXR activation. H-P cells were transfected with CYP3A4 promoter reporter and cotreated with 10 µM rifampicin (RIF) and increasing concentrations of the indicated compounds (**B**) or treated with increasing concentrations of the novel compounds only (**C**) for 24 h. Data are expressed as mean ± SD fold induction with respect to the DMSO-treated cells from three independent experiments. IC_50_ and EC_50_ values calculated with nonlinear regression using formula with 3 parameters (constraint bottom =1) using GraphPadPrism.

We applied a virtual screening approach to identify potential PXR ligands from the TüKIC compound library (Figure 1A). Based on the in silico evaluation, the candidate ligands were tested in antagonist and agonist modes using the PXR-dependent CYP3A4 reporter gene assay (Supplementary Figure S1). This initial screen, complemented by two subsequent structural analog searches based on the assay results (Figure 1A), resulted in the identification of four novel potential PXR antagonists, which inhibited induction of CYP3A4 reporter activity by the prototypical agonist rifampicin by more than 50% (Figure 1B and Figures S1–S3). The most potent compounds **73** and **100** demonstrated 60–70% inhibition of rifampicin-mediated induction, while only weakly activating the CYP3A4 reporter on their own, at about 10% of rifampicin activity (Supplementary Figures S2 and S3, respectively). Inhibition of agonist-induced PXR activation was not rifampicin-specific, as all novel compounds, except **12**, suppressed SR12813-induced PXR activation to a similar extent as rifampicin-induced activation (Supplementary Figure S4). With the single exception of **12**, the identified candidate ligands elicited pronounced toxicity at 30 µM with cell viability of ≤50%. At 10 µM, modest toxicity was observed, with residual viability between 75 to 83% (Supplementary Figure S5). Thus, maximum concentrations were limited to 10 µM in the subsequent concentration–response analyses. Only **12** was used up to 30 µM. Results of the concentration–response analyses, with opposing effects of the compounds in agonist and antagonist modes (Figure 1B,C), further argue against a significant influence of the limited cytotoxicity at 10 µM on PXR-dependent reporter activity. Compounds **73** and **100** showed IC_50_ values of 8.3 µM (95% CI 6.1–11.7 µM) and 2.8 µM (95% CI 2.1–3.8 µM), respectively. Thereby these compounds demonstrated stronger inhibition of rifampicin-induced PXR activation compared to **2** and **12**, which displayed IC_50_ values of 11.2 µM (95% CI 7.7–17.2 µM) and 33.7 µM (95% CI 20.2–61.6 µM), respectively (Figure 1B). As concentrations higher than 10 µM or 30 µM (compound **12** only) could not be used, IC_50_ values were calculated from concentration–response analyses, which did not result in a lower plateau, thereby limiting the significance of the absolute values. Furthermore, **73** and **100** activated PXR only weakly, with maximal effects (E_max_) of about 2-fold, but with low EC_50_ values of 0.41 µM (95% CI 0.18–0.87 µM) and 0.15 µM (95% CI 0.04–0.45 µM), respectively (Figure 1C). On the other hand, concentration–response curves of **2** and **12** did not reach plateau at the maximum concentration of 10 µM and 30 µM, respectively. Therefore, the observed E_max_ and calculated EC_50_ values must be interpreted with caution. This is also reflected by the inability to calculate the complete confidence interval for the EC_50_ of **2** (EC_50_ of 7.0 µM, 95% CI 1.2–??? µM) and by the large confidence interval with **12** (EC_50_ of 22.3 µM, 95% CI 8.8–110 µM). Interestingly, we identified also a structurally related strong activator, **109** (Figure 1A and Figure S3), which showed 27-fold maximal induction and EC_50_ of 44 µM (95% CI 20–??? µM) (Figure 1C). Due to the incomplete confidence interval calculation, EC_50_ of **109** has to be interpreted with caution.

**Figure 2:**
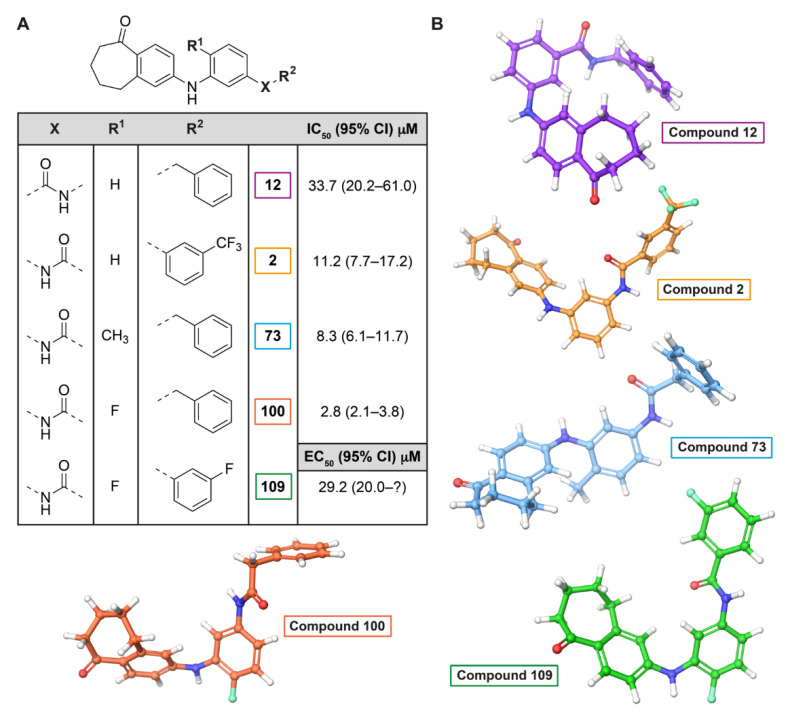
Structure–activity relationship of the identified compounds and their preferred conformations. (**A**) All compounds share a common phenylaminobenzosuberone core, displaying structural differences in X, R^1^, and R^2^ positions. (**B**) The lowest energy conformation of the compounds in water based on QM Conformer predictor (see details in Methods and Supplementary Table S1 and Supplementary Figures S6–S10).

The identified hit compounds share a common phenylaminobenzosuberone scaffold (Figure 2A). Compound **12** has an inverted amide linking the R^2^ and is showing the highest IC_50_-value. The length of the R^2^ seems also important for PXR inhibition, as the shorter phenyl containing **109** does not inhibit PXR. Of note, **2**, which also has a phenyl group in its R^2^, has an additional sterically large trifluoromethyl substituent in its phenyl ring. Furthermore, compounds with the highest inhibitory activity display a heavier substituent than H in their R^1^. Based on the lowest energy conformations in water (Figure 2B, Supplementary Table S1, Supplementary Figures S6–S10), the most potent inhibitors **73** and **100** appear to prefer a more extended conformation, while the other compounds tend to appear in a more folded configuration. The heavier R^1^-substituent found in **73** and **100** seems to promote the extended conformations (100% and 84%, respectively), while in **12**, with an H-atom in this position, this conformation is not preferred (11%) (Supplementary Table S1, Supplementary Figures S6–S10).

### 3.2. Compounds 73 and 100 Demonstrate Competitive Antagonism of PXR

**Figure 3:**
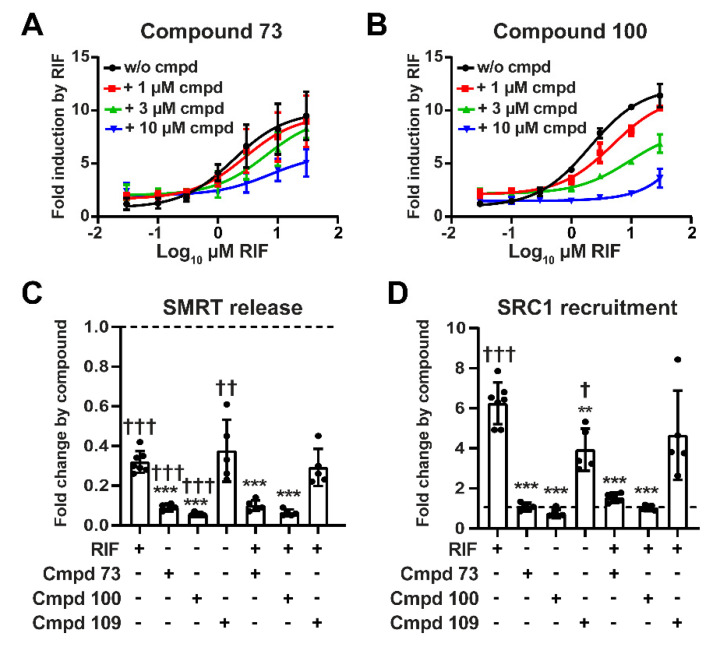
Compounds **73** and **100** act as mixed competitive/noncompetitive passive antagonists of PXR. (**A**,**B**) Effects of novel PXR antagonists on the concentration–response curve of rifampicin. H-P cells were transfected with CYP3A4 promoter reporter and cotreated with increasing concentrations of rifampicin (RIF) with or without fixed concentrations of **73** and **100**. Effects of novel compounds on PXR‘s interactions with corepressor SMRT (**C**) and coactivator SRC1 (**D**). HepG2 cells were cotransfected with GL4-G5 reporter gene and expression plasmids encoding VP16-PXR LBD(108–434) and (**C**) GAL4-DBD-SMRT-RID or (**D**) GAL4-DBD-SRC1-RID fusion proteins and treated with 0.2% DMSO, 10 μM test compounds alone, or cotreated with 10 μM rifampicin. Data are shown as mean ± SD fold induction with respect to DMSO-treated cells from five independent experiments and individual experiments illustrated with dots. Statistically significant differences are illustrated with asterisks or daggers. ** *p* < 0.01, *** *p* ˂ 0.001, compared to 10 μM rifampicin-treated cells analyzed by one-way ANOVA with Dunnett’s multiple comparisons test. † *p* < 0.05, †† *p* ˂ 0.01, and ††† *p* ˂ 0.001 single treatments compared to DMSO, which was set as 1, analyzed by one sample *t*-test corrected by the method of Bonferroni.

To investigate the mechanism of PXR inhibition by the two strongest inhibitors **73** and **100**, their effects on the concentration–response curve of rifampicin were assessed. Figure 3A shows that at lower concentrations (1 and 3 µM) compound **73** induced a parallel dextral shift of the rifampicin concentration–response curve with corresponding increases in the EC_50_ values of rifampicin. Without compound **73**, EC_50_ of rifampicin was 1.8 µM (95% CI 0.76–4.5 µM), which increased in the presence of 1 µM or 3 µM of compound **73** to 2.9 µM (95% CI 1.0–9.1 µM) or 6.4 µM (95% CI 2.5–19.6 µM), respectively. With 10 µM of compound **73**, rifampicin EC_50_ was further elevated to 7.4 µM (95% CI 2.0–47.7 µM), and reduction of E_max_ was pronounced. Compound **100** also caused a dextral shift of the rifampicin concentration–response curve with corresponding increases in the EC_50_ values of rifampicin. EC_50_ value of rifampicin raised from 2.0 µM (95% CI 1.6–2.6 µM) in the absence of **100** to 4.8 µM (95% CI 3.4–7.0 µM) with 1 µM of **100**, and further to 9.3 µM (95% CI 5.4–17.0 µM) with 3 µM of **100** (Figure 3B). Rifampicin EC_50_ in the presence of 10 µM **100** could not be calculated reliably. These observations indicate that compounds **73** and **100** act as competitive antagonists of PXR. The reduction in E_max_, which was observed especially at the highest test concentration of both compounds, may indicate additional noncompetitive antagonism.

### 3.3. Compounds 73 and 100 Disrupt PXR’s Coregulatory Protein Interactions

We assessed the effect of the novel antagonists and, for comparison, of the activator **109** on the ligand-dependent interactions of PXR with coregulatory proteins using respective mammalian two hybrid assays. Rifampicin, the novel antagonists **73** and **100**, as well as the activator **109**, all impaired the constitutive interaction of PXR with the corepressor silencing mediator of retinoic acid and thyroid hormone receptor (SMRT, NCOR2) (Figure 3C). Release of SMRT from PXR was even more pronounced by compounds **73** and **100** than by rifampicin. As expected, rifampicin induced the interaction of PXR with the coactivator steroid receptor coactivator 1 (SRC1, NCOA1). Similarly, PXR‘s interaction with SRC1 was induced by the activating compound **109** (Figure 3D). In contrast, compounds **73** and **100** did not induce any interaction of PXR with coactivator SRC1. Furthermore, they abrogated the rifampicin-dependent recruitment of SRC1 by PXR. These results indicate that **73** and **100** can be classified as passive antagonists as they impair the interactions of PXR with both coactivators and corepressors [40], while **109** exhibits agonist properties.

### 3.4. Phenylaminobenzosuberones Demonstrate Direct Binding to the PXR-LBP

**Figure 4:**
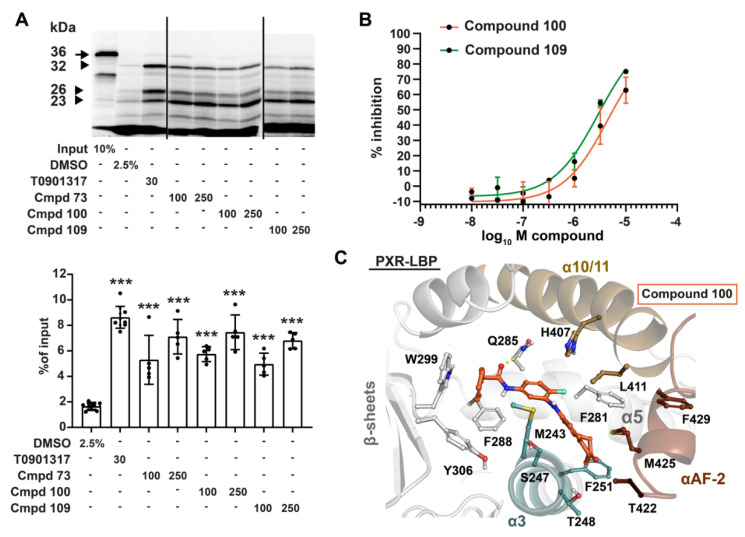
Phenylaminobenzosuberones demonstrate direct binding to PXR. (**A**) Limited proteolytic digestion assay was conducted by preincubating ^35^S-labeled PXR LBD with 100 μM or 250 μM test compounds, 30 μM T0901317, or 2.5% DMSO. Upper panel shows the radioluminographic scan of a representative experiment. Arrow shows 36 kDa input of PXR LBD, and arrow heads show protected 32, 26, and 23 kDa fragments after limited proteolytic digest with trypsin. Lower panel shows respective densitometric quantifications of the sum of the three protected fragments. Columns show mean ± SD of five independent experiments (each performed as single measurement) and the individual experiments illustrated with dots, calculated with respect to input. Statistically significant differences are illustrated with asterisks. *** *p* ˂ 0.001, compared to respective preincubations with DMSO and analyzed by one-way ANOVA with Dunnett’s multiple comparisons test. (**B**) Competitive radioligand binding assay demonstrates direct binding of compounds **100** and **109** to PXR. Data are shown as means ± SD of % inhibition of control specific binding by test compounds from duplicate analysis. (**C**) Putative binding mode of compound **100** in the PXR-LBP. A representative snapshot taken from the microsecond timescale MD simulations [42] is shown in the figure, where compound **100** (orange C-atoms stick model) is shown with the closely located key residues of PXR-LBP (shown with sticks, with colors matching to their secondary structure region: αAF-2, dark brown; α3-helix, cyan; α10/11-helix, light brown; other, white). The benzyl moiety of **100** is occupying the hydrophobic subpocket formed by F288, W299, and Y306, while the benzosuberone is oriented toward the αAF-2 region. H-bond to Q285 from the amide is shown with yellow dashed line.

Ligands of nuclear receptors physically interact with the respective ligand binding domain (LBD). To confirm the binding of the antagonists **73** and **100**, as well as of the activator **109**, to the PXR LBD, the respective in vitro limited proteolytic digestion assay was applied [37]. The assay relies on conformational changes in the LBD that are induced by the ligand binding to it, which alter the accessibility of proteases to cleavage sites [41]. Figure 4A shows that all compounds, similar to the reference ligand T0901317, resulted in increased protection of three proteolytic fragments from digestion, albeit at relative intensities different from T0901317. These data indicate that the novel compounds act as ligands of PXR. Due to the nature of the PXR limited proteolytic digestion in vitro assay, it required higher concentrations of the ligands than PXR activation in cellular assays, which has been described previously [37].

To corroborate these results, the binding of compounds **100** and **109** was further analyzed by competitive radioligand binding assays. Figure 4B shows that compounds **100** and **109** inhibited the binding of ^3^H-labeled SR12813 to PXR with IC_50_ of 4.5 µM (95% CI 1.9–15.4 µM) and 2.9 µM (95% CI 1.9–4.7 µM), respectively. We also investigated the binding of compound **100** by microsecond timescale molecular dynamics (MD) simulations [42]. Figure 4C illustrates one putative binding configuration of compound **100** in the ligand binding pocket (LBP) of PXR based on the simulation data. In conclusion, the results of the limited proteolytic digestion assay and competitive radioligand binding assay, supported by MD simulations, indicate a physical interaction of the novel compounds with PXR and thus confirm ligand binding.

**Figure 5:**
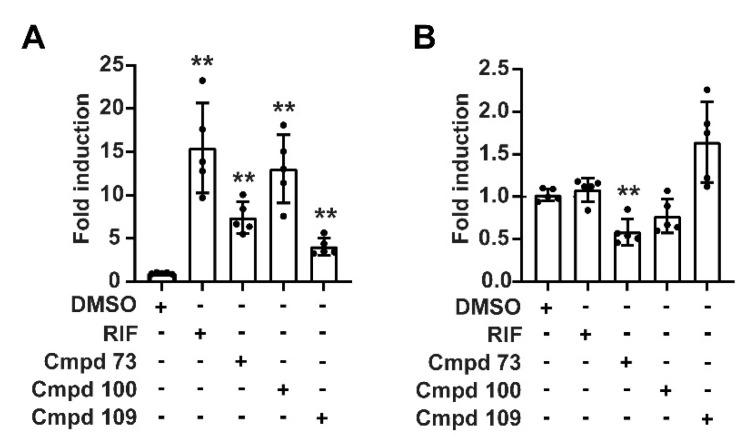
PXR binding of phenylaminobenzosuberones is indicated by cellular PXR LBD assembly assay and inhibition of LBP-filled PXR mutant (**A**) Novel compounds induce PXR LBD assembly. HepG2 cells were transiently transfected with GL4-G5 reporter, GAL4-DBD/PXR LBD(132–188), and VP16-AD/PXR LBD(189–434) fusion protein expression plasmids and treated with 0.1% DMSO, 10 μM rifampicin, or 10 μM test compounds for 24 h. Data are expressed as mean ± SD fold induction with respect to DMSO-treated cells from five independent experiments and individual experiments illustrated with dots. (**B**) Effects of novel compounds on LBP-filled PXR mutant. HepG2 cells were transiently transfected with CYP3A4 reporter and expression plasmid encoding PXR(S208W/S247W/C284W) and treated as described above. Data are shown as mean ± SD fold induction, with respect to treatment with DMSO only, from five independent experiments. Statistically significant differences are illustrated with asterisks. ** *p* ˂ 0.01, compared to respective treatments with DMSO analyzed by repeated measures one-way ANOVA with Dunnett’s multiple comparisons test.

As a cellular assay equivalent of the biochemical ligand binding assays, we applied the mammalian two-hybrid LBD assembly assay, which identifies both agonists and antagonists of nuclear receptors [43]. Figure 5A shows that antagonists **73** and **100** and agonist **109** induced the assembly of the PXR LBD. PXR antagonism can occur either by competitive binding into the PXR ligand binding pocket (LBP), which has been demonstrated with SPA70 [44], or by binding exclusively or additionally to an allosteric site outside LBP. Exclusive binding outside the LBP has been observed with ketoconazole and camptothecin [45,46], while coumestrol and pimecrolimus demonstrated binding both to the LBP and to an allosteric site outside it [16,47]. As the previous assays do not distinguish between these possibilities, we next investigated the effects of the novel compounds on the constitutive activity of a LBP-filled PXR mutant. As a result of mutation, ligand binding into the pocket is prevented, and the mutant exhibits high constitutive activity [47]. While **100** did not affect constitutive activity of the PXR mutant, **73** still suppressed the respective activity by 40% (Figure 5B). These data suggest additional binding of **73** outside the LBP, at least with the LBP-filled mutant, which further supports the above suggested noncompetitive antagonism for **73** (see Figure 3A). In contrast, the allosteric binding of compound **100** to PXR outside the LBP is not supported by this assay.

### 3.5. Kinase Inhibition Profile of 100 and 109

Several kinases have shown to phosphorylate PXR and thereby to modulate the transcriptional activity of the receptor [48,49,50,51,52]. Usually, phosphorylation of human PXR results in impaired transcriptional activity and repression of target gene expression. Thus, kinase inhibition may contribute indirectly to the activation of PXR. Compounds **100** and **109**, as representatives of structurally related antagonists and agonist, were tested against a set of 335 wild-type kinases to disclose their kinase inhibition profile (Supplementary Data S1). At 10 µM, compound **100** inhibited five kinases, namely BRAF, MAPKAPK3, p38β, PKA, and RAF1 by ≥50%. At 1 µM, only RAF1 was inhibited considerably. With 10 µM of compound **109**, eight kinases, BRAF, CK1-δ, CK1-ε, CK1-γ3, p38α, p38β, PKA, and RAF1, were inhibited at least by 50%. CK1-δ, p38α and RAF1 were already inhibited to this extent at 1 µM. Of these kinases, only PKA was previously shown to affect PXR function by inhibitory phosphorylation [49]. Consequently, the inhibition of PKA, which could promote PXR activation, might explain the observed limited PXR activation occurring with **100,** and, on the other hand, it may contribute to the strong PXR activation by **109**.

### 3.6. Nuclear Receptor Selectivity of Novel PXR Ligands

**Figure 6:**
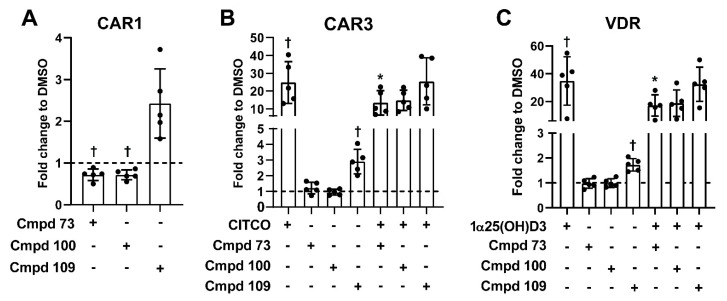
Selectivity of novel PXR ligands within the NR1I group of nuclear receptors. HepG2 cells were transiently transfected with CYP3A4 reporter and expression plasmids encoding (**A**) CAR1 or (**B**) CAR3, or (**C**) direct repeat (DR3)_3_ reporter and expression plasmid encoding VDR. Cells were treated with 0.2% DMSO or 10 µM chemicals as indicated. 1α,25(OH)_2_D_3_ was used at 1 µM. Data are shown as mean ± SD fold induction with respect to DMSO-treated cells from five independent experiments and individual experiments illustrated with dots. Statistically significant differences are illustrated with asterisks and daggers. † *p* ˂ 0.05 single treatments compared to DMSO-treated cells set as 1 and analyzed by one sample *t*-test corrected by the method of Bonferroni. * *p* < 0.05 cotreatments compared to (**B**) CITCO or (**C**) 1α,25(OH)_2_D_3_ analyzed by repeated measures one-way ANOVA with Dunnett’s multiple comparisons test.

To assess the selectivity of the novel PXR ligands, we determined their effects on CAR- and VDR-mediated transactivation. CAR and VDR belong to the same NR1I group of nuclear receptors as PXR and share 37–45% sequence similarity in their LBD [53]. Both **73** and **100** slightly suppressed the constitutive activity of isoform CAR1 by 30% (Figure 6A). Compound **73** also impaired the ligand-induced activation of CAR3 by CITCO by 46% (Figure 6B) and reduced the ligand-induced VDR activation by 51% (Figure 6C), while **100** displayed no respective effects. Neither CAR3 nor VDR were activated by **73** or **100** on their own. In contrast, the agonist **109** weakly activated both CAR3 and VDR, but the effects were only 12% and 5% of the effects of the respective prototypical ligands CITCO and 1α,25-dihydroxy vitamin D_3_. Activation of CAR1 by compound **109** did not reach statistical significance after Bonferroni correction, but a trend was observed (*p* = 0.0552). These results indicate that the novel PXR antagonists, and here especially **100**, demonstrate only minor inhibitory effects on CAR and VDR.

### 3.7. Expression of Prototypical Endogenous PXR Target Genes Is Differentially Modulated by the Compounds

**Figure 7:**
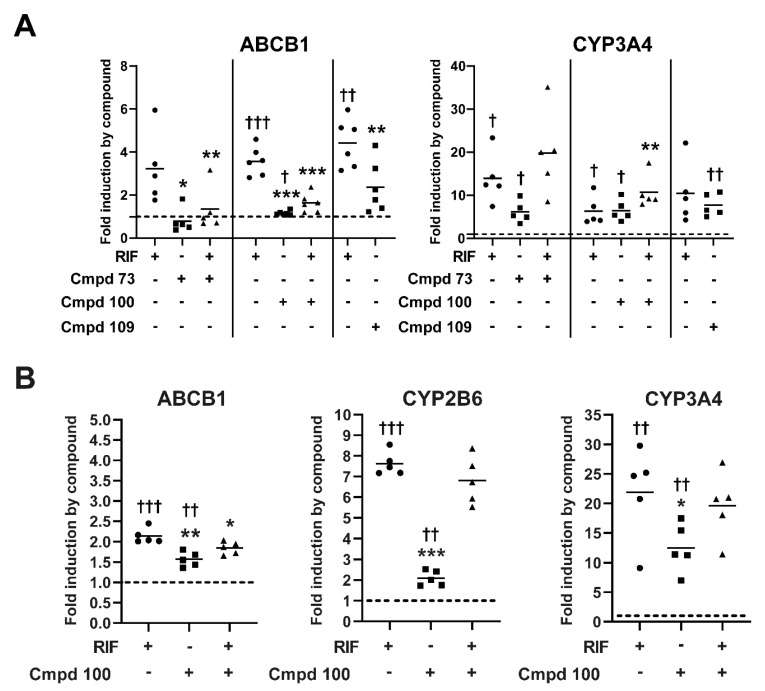
Effects of novel PXR ligands on endogenous PXR target gene expression. (**A**) LS174T cells were treated with 0.1–0.2% DMSO, 10 µM rifampicin (RIF), or 10 µM test compounds or cotreated with 10 µM RIF and compounds **73** and **100** for 48 h (**109**) or 72 h (**73**, **100**). mRNA expression was determined by RT-qPCR and normalized to the expression of 18S rRNA. Data are expressed as mean fold induction with respect to DMSO-treated cells, expression in which was set as 1, from six independent experiments and individual experiments illustrated with dots. (**B**) HepaRG cells were treated with 0.2% DMSO, 10 µM RIF, or 10 µM compound **100** or cotreated with 10 µM RIF and compound **100** for 48 h. mRNA expression was measured as described above. Data are expressed as mean fold induction with respect to DMSO-treated cells from five independent experiments and individual experiments illustrated with dots. Statistically significant differences are illustrated with asterisks and daggers. * *p* ˂0.05, ** *p* < 0.01, and *** *p* ˂ 0.001 compared to 10 μM rifampicin-treated cells analyzed by repeated measures one-way ANOVA with Dunnett’s multiple comparisons test (**73**, **100**) or by paired *t*-test (**109**). † *p* ˂ 0.05, †† *p* ˂ 0.01, and ††† *p* < 0.001 single treatments compared to DMSO, which was set as 1, analyzed by one sample *t*-test corrected by the method of Bonferroni.

To investigate the effects of the novel PXR ligands on the expression of endogenous PXR target genes, we utilized LS174T colorectal carcinoma cells, the PXR expression level of which is comparable to liver [31]. In these cells, rifampicin induced the expression of ABCB1 and CYP3A4. Expression of ABCB1 was not induced by **73** and **100**, and these compounds also suppressed its rifampicin-mediated induction (Figure 7A). Despite being characterized as PXR antagonists, both compounds induced CYP3A4 expression, which was comparable to induction by rifampicin. In accordance with its agonist properties, **109** induced both genes, whereby ABCB1 was induced to a lesser extent than by rifampicin. These results indicate that compounds **73** and **100** antagonize PXR activity in a gene-specific manner in intestinal carcinoma cells.

To investigate whether PXR antagonism can be observed also in other tissues, the effect of compound **100** on PXR target gene expression was analyzed in differentiated HepaRG cells, which closely resemble functional hepatocytes [54]. Compound **100** induced the expression of ABCB1, CYP2B6, and CYP3A4 (Figure 7B). However, the induction of these three genes was considerably weaker than by the prototypical agonist rifampicin. In contrast to LS174T cells, compound **100** demonstrated only minor inhibitory effects on rifampicin-induced expression of ABCB1. In summary, these PXR target gene expression data indicate that compound **100** might possess partial agonist activity in hepatic cells.

## 4. Discussion

By a combination of in silico molecular modeling and cellular PXR-dependent reporter gene assays, we identified four novel PXR inhibitors and a structurally related full agonist from the in-house TÜKIC compound library. Comprehensive subsequent analyses, addressing key features of nuclear receptor biology, confirmed the most potent inhibitors **73** and **100** as PXR ligands, demonstrating passive, mixed competitive/noncompetitive antagonism, and gene- and tissue-specific modulation of PXR target gene expression, qualifying them as selective PXR modulators.

The lack of experimental structural data for a PXR–antagonist complex, combined with the promiscuous nature of PXR ligand binding, brings challenges to identify antagonists by docking [55], which as a method has limitations on its own [56]. Although, we were able to identify competitive and mixed competitive/noncompetitive antagonists here starting from an in silico screen essentially relying on molecular docking, long timescale MD simulations are required for a proper estimation of the antagonist compound binding mode. A detailed analysis of compound **100** binding mode in the PXR LBD is provided in a complementary publication [42].

The identified antagonists share a common phenylaminobenzosuberone scaffold with the agonist **109**. The high structural similarity of antagonists and the agonist demonstrates that even subtle structural changes have great impact on PXR activation and inhibition. The only obvious conformational difference was that the most potent antagonists **73** and **100** appeared to prefer more extended conformations. We and others have observed previously that subtle structural changes can completely change the activity of PXR ligands. For example, the reduction of artemisinin to its lactol derivative dihydroartemisinin abrogates PXR ligand binding in the PXR-LBD assembly assay [57]. More recently, Li et al. demonstrated for the PXR antagonist SPA70 that small alterations in substituents at the common scaffold change the activity of the compound from antagonistic to full agonistic [58]. Our findings here emphasize the challenging design of PXR antagonists due to the great impact of subtle structural changes to PXR activity.

Regarding characterization of the respective mechanism of antagonism, we focused on the two strongest compounds **73** and **100**. According to the operational definition of antagonism, the dextral shift in the concentration–response curve of PXR agonist rifampicin by increasing concentrations of inhibitors suggested classification of the novel compounds as competitive antagonists. However, as the maximal effect was also decreasing, contribution of a noncompetitive/allosteric component in antagonism appears obvious also. If the compounds act as competitive antagonists, they have to bind into the LBP of PXR. Results from different assays provided independent evidence for LBP binding of the compounds. First, both **73** and **100** demonstrated displacement of LBP-bound agonist SR12813 in the competitive radioligand binding assay. Second, MD simulations enabled the identification of the putative binding mode of compound **100** in the LBP of PXR. Third, the compounds induced the assembly of the PXR LBD, which is not achieved by antagonists with exclusive allosteric binding, such as camptothecin and pazopanib [16]. Independent evidence for noncompetitive or allosteric binding outside the LBP was obtained only for **73**, which still demonstrated limited inhibition of the constitutive activity of the LBP-filled PXR triple mutant S208W/S247W/C284W. Only exclusive allosteric antagonists, such as camptothecin and pazopanib [16] or mixed competitive/allosteric antagonists, such as coumestrol [47] and pimecrolimus [16], showed inhibition of this mutant’s activity. Compounds demonstrating only competitive PXR antagonism, such as nelfinavir, did not [59].

Passive antagonism is suggested by the fact that the novel antagonists abolished the rifampicin-induced interaction of PXR with coactivator SRC1, as well as impairing the constitutive interaction of PXR with corepressor SMRT. Except SPA70, for which recruitment of SMRT was shown [44], previously described PXR antagonists, such as ketoconazole, camptothecin, sulforaphane, pazopanib, and pimecrolimus, all demonstrated the same effects on coregulator interaction as described here for **73** and **100** [16,46]. Molecular dynamic simulations of compound **100** indicated that binding it affects the conformation of the PXR LBD in distinct regions, including the AF-2 region (where coactivators and corepressors bind), supporting the observed biological results [42].

The novel antagonists **73** and **100** demonstrated gene-specific effects in LS174T colorectal cancer cells, not inducing on their own the expression of ABCB1, but inducing CYP3A4 expression to a similar extent as rifampicin. Furthermore, both compounds antagonized exclusively the rifampicin-mediated induction of ABCB1. The strong induction of CYP3A4 and absence of antagonism of the rifampicin-dependent activation is surprising, as the compounds were identified by their inhibition of rifampicin-mediated CYP3A4 enhancer/promoter activation. However, in contrast to the transfected reporter gene construct, the regulatory region of the endogenous gene resides in chromatin. Differences in the promoter context between genes, resulting in altered interaction with or altered activity of coregulators, have been suggested for the explanation of gene-specific effects of nuclear receptor ligands [60]. Alternatively, the observed induction of endogenous CYP3A4 expression in LS174T cells might not result from PXR agonism itself. Given the fact that **100** was shown to inhibit PKA at 10 µM, it is conceivable that inhibition of the PXR-inhibitory kinase activity of PKA may participate in CYP3A4 induction, especially as it was shown that the activation of PKA resulted in repression of CYP3A4 expression [49]. Even in this scenario, we would have to assume that the effect of PKA inhibition on PXR activity is not relevant for ABCB1. The observed tissue-specific effects of compound **100** might result from divergent coactivator versus corepressor levels, varying PXR levels, or different activities of coactivators or PXR due to modulation of kinase signaling by the compound. These potential cellular variables have been shown previously to determine the activity of selective modulators of nuclear receptors [60].

With BRAF, RAF1, p38β, and MAPKAPK3, four of the five kinases, which were strongly inhibited by **100**, belong to the MAPK/ERK pathway. Hitherto and in conventional 2D cell culture, the MAPK/ERK pathway has not been associated with regulation of hepatic cytochrome P450 expression, as its inhibition by dominant-negative MEK1 did not induce CYP3A4 in HepG2 cells [14]. However, it was recently shown in 3D spheroid cultures of primary human hepatocytes that pharmacological inhibition of the pathway induced CYP3A4 and CYP2B6 expression [61]. By siRNA-mediated knockdown of PXR, the authors further showed that PXR is involved in the respective CYP3A4 and CYP2B6 induction. Consequently, they suggested inhibition of PXR by the MAPK/ERK pathway. Here we used differentiated HepaRG cells in 2D culture, which are not a pure hepatocyte culture but also contain biliary epithelial cells [54]. If differentiated HepaRG cells resembled 3D hepatocyte spheroids in terms of MAPK/ERK pathway activity, it is conceivable that treatment with **100** might result in inhibition of this pathway, followed by release of PXR inhibition and consequently CYP3A4 and CYP2B6 induction. In this case, the induction of CYP3A4 and CYP2B6 by **100** would not indicate PXR agonism but indirect activation through MAPK/ERK pathway inhibition. Further research is required to distinguish between these possibilities.

Additional inhibition of PXR by protein kinase inhibitors may provide benefit for cancer therapy in several ways. First, many kinase inhibitors are metabolized by cytochrome P450 enzymes and transported by MDR1/P-glycoprotein [62,63,64,65,66], the encoding genes, which are regulated by PXR. In addition, at least 10 of the roughly 70 approved kinase inhibitors used for the treatment of cancer have even been shown to activate PXR [13,15,63,64,65,67] (Supplementary Table S2), which may result in autoinduction of drug metabolism and elimination. Thus, generating derivatives, which inhibit the receptor, may result in reduced drug metabolism and/or drug elimination, which may improve drug efficacy and reduce off-target toxicity as allowing lower dosing. The feasibility of a respective structure-based synthesis approach has recently been demonstrated for B-RAF inhibitors structurally related to the PXR activator dabrafenib, which neither bind to nor activate the receptor [68]. Second, activation of PXR is known to promote cancer cell growth and to contribute to the development of cancer drug resistance [8,9]. Strategically, a dual PXR/protein kinase inhibitor is expected to target tumor growth by two different mechanisms and concomitantly will prevent generation of PXR-dependent chemoresistance. The clinical relevance of the concept is illustrated by PXR mediating the chemoresistance of hepatocellular carcinoma to the multikinase inhibitor sorafenib [69].

In conclusion, we have identified and characterized novel selective receptor modulators of PXR from an in-house kinase inhibitor compound library. Their common phenylaminobenzosuberone scaffold represents a previously unknown PXR ligand structure and may be used as a starting point for the synthesis of the suggested dual PXR and protein kinase inhibitors.

## Data Availability

All data are included in the figures in the manuscript and as supplementary data.

## Supplementary Materials

The following supporting information can be downloaded at: https://www.mdpi.com/article/10.3390/cells11081299/s1: Supplementary information file: Supplementary methods and compound NMR spectra; Supplementary Figure S1: Effects of 56 in silico screened TüKIC compounds (A) alone or (B) in combination with 10 μM rifampicin on PXR-mediated transactivation of CYP3A4 reporter gene; Supplementary Figure S2: Effects of first round structural analogues (A) alone or (B) in combination with rifampicin on PXR-mediated transactivation of CYP3A4 reporter gene; Supplementary Figure S3: Effects of second round structural analogues (A) alone or (B) in combination with rifampicin on PXR-mediated transactivation of CYP3A4 reporter gene; Supplementary Figure S4: Effects of potential novel PXR antagonists in combination with 1 μM SR18213 on PXR-mediated transactivation of CYP3A4 reporter gene; Supplementary Figure S5: Cell viability of HepG2 cells following 24 h treatment with potential novel PXR ligands; Supplementary Figure S6: QM Conformer & Tautomer Predictor output conformations of compound **2**; Supplementary Figure S7: QM Conformer & Tautomer Predictor output conformations of compound **12**; Supplementary Figure S8: QM Conformer & Tautomer Predictor output conformations of compound **73**; Supplementary Figure S9: QM Conformer & Tautomer Predictor output conformations of compound **100**; Supplementary Figure S10: QM Conformer & Tautomer Predictor output conformations of compound **109**; Supplementary Table S1: QM Conformer & Tautomer Predictor output conformations and their energies; Supplementary Table S2: PXR activating protein kinase inhibitors. Supplementary Data File S1: Kinase inhibition profile of compounds **100** and **109**. Supplementary Data File S2: 3D coordinates of the conformations of the small-molecules. Supplementary Data File S3: 3D coordinates of the PXR LBD compound 100 putative binding mode.
