# Supplementary material for: Target Hopping from Protein Kinases to PXR: Identification of Small-Molecule Protein Kinase Inhibitors as Selective Modulators of Pregnane X Receptor from TüKIC Library
Source: Cells. 2022 Apr 12;11(8):1299. doi: 10.3390/cells11081299 (PMC9030254; doi:10.3390/cells11081299)
Supplement: Supplementary file 1 [file cells-11-01299-s001.zip › Supplementary_Data_file_1.pdf]

Profiling of compounds at two concentrations each against 335 wild-type protein kinases; singlicate measurement

Residual activities (% of control)

Mean residual activity < 50 %

#	Kinase Name	Kinase Family*	Cpd ID / Assay Conc. (M)	Cmpd 100	Cmpd 100	Cmpd 109	Cmpd 109	
				1.0E-06	1.0E-05	1.0E-06	1.0E-05	
1	ABL1	TK		90	88	91	91	
2	ABL2	TK		97	93	95	92	
3	ACK1	TK		112	92	96	89	
4	ACVR1	TKL		97	93	88	97	
5	ACVR1B	TKL		92	90	98	87	
6	ACVR2A	TKL		89	95	99	108	
7	ACVR2B	TKL		95	169	104	167	
8	ACVRL1	TKL		106	100	94	109	
9	AKT1	AGC		99	88	101	97	
10	AKT2	AGC		92	82	92	92	
11	AKT3	AGC		114	100	95	102	
12	ALK	TK		86	72	95	92	
13	AMPKalpha1	CAMK		112	101	99	98	
14	ARK5	CAMK		90	91	92	88	
15	ASK1	STE		87	85	87	88	
16	AuroraA	OTHER		96	91	93	90	
17	AuroraB	OTHER		101	90	101	85	
18	AuroraC	OTHER		110	101	99	101	
19	AXL	TK		103	99	98	93	
20	BLK	TK		118	110	82	82	
21	BMPR1A	TKL		101	82	92	88	
22	BMPR1B	TKL		102	121	101	128	
23	BMX	TK		111	71	100	83	
24	BRAF	TKL		58	25	67	39	
25	BRK	TK		105	106	105	84	
26	BRSK1	CAMK		101	91	92	92	
27	BRSK2	CAMK		113	99	109	100	
28	BTK	TK		99	78	99	91	
29	BUB1B	OTHER		108	96	104	106	
30	CAMK1D	CAMK		85	88	93	89	
31	CAMK2A	CAMK		96	94	95	91	
32	CAMK2B	CAMK		144	115	132	104	
33	CAMK2D	CAMK		103	90	92	88	
34	CAMK2G	CAMK		105	107	109	111	
35	CAMK4	CAMK		102	97	97	89	
36	CAMKK1	OTHER		78	80	77	75	
37	CAMKK2	OTHER		97	95	84	81	
38	CDC42BPA	AGC		114	110	103	116	
39	CDC42BPB	AGC		112	107	106	110	
40	CDC7/DBF4	OTHER		98	93	96	91	
41	CDK1/CycA2	CMGC		85	83	86	88	
42	CDK1/CycB1	CMGC		107	100	106	114	
43	CDK1/CycE1	CMGC		102	100	96	98	
44	CDK12/CycK	CMGC		104	100	105	99	
45	CDK13/CycK	CMGC		88	84	88	92	
46	CDK16/CycY	CMGC		99	101	96	100	
47	CDK17/p35NCK	CMGC		102	84	109	92	
48	CDK18/CycY	CMGC		93	88	91	98	
49	CDK19/CycC	CMGC		81	77	83	74	
50	CDK2/CycA2	CMGC		87	106	83	94	
51	CDK2/CycD1	CMGC		102	119	106	87	
52	CDK2/CycE1	CMGC		86	118	93	103	
53	CDK20/CycH	CMGC		103	98	110	121	
54	CDK20/CycT1	CMGC		98	92	96	101	
55	CDK3/CycC	CMGC		97	89	87	96	
56	CDK3/CycE1	CMGC		90	80	91	82	
57	CDK4/CycD1	CMGC		104	94	93	108	
58	CDK4/CycD2	CMGC		94	86	94	91	
59	CDK4/CycD3	CMGC		93	89	94	89	
60	CDK5/p25NCK	CMGC		99	90	95	95	
61	CDK5/p35NCK	CMGC		105	93	95	101	
62	CDK6/CycD1	CMGC		93	89	84	90	
63	CDK6/CycD2	CMGC		97	97	105	94	
64	CDK6/CycD3	CMGC		90	92	87	93	
65	CDK7/CycH/MAT1	CMGC		94	83	89	92	
66	CDK8/CycC	CMGC		82	60	79	61	
67	CDK9/CycK	CMGC		91	89	90	95	
68	CDK9/CycT1	CMGC		95	89	92	93	
69	CHK1	CAMK		91	91	88	90	
70	CHK2	CAMK		92	72	94	74	
71	CK1alpha1	CK1		112	108	108	102	
72	CK1delta	CK1		110	106	23	15	
73	CK1epsilon	CK1		107	117	52	15	
74	CK1gamma1	CK1		111	115	86	57	
75	CK1gamma2	CK1		104	114	84	70	
76	CK1gamma3	CK1		101	105	79	32	
77	CK2alpha1	OTHER		102	106	102	98	
78	CK2alpha2	OTHER		137	134	119	118	
79	CLK1	CMGC		99	79	105	96	
80	CLK2	CMGC		117	107	112	103	
81	CLK3	CMGC		130	114	110	105	
82	CLK4	CMGC		103	98	105	108	
83	COT	STE		93	106	95	97	
84	CSF1R	TK		100	96	103	94	
85	CSK	TK		106	96	112	109	
86	DAPK1	CAMK		127	110	109	102	
87	DAPK2	CAMK		113	124	110	133	
88	DAPK3	CAMK		105	119	108	107	
89	DCAMKL2	CAMK		101	89	100	103	
90	DDR2	TK		94	71	93	64	
91	DMPK	AGC		101	100	99	94	
92	DNAPK	ATYPICAL		95	97	96	106	
93	DYRK1A	CMGC		92	98	92	92	
94	DYRK1B	CMGC		103	117	103	96	
95	DYRK2	CMGC		89	91	98	92	
96	DYRK3	CMGC		98	104	96	88	
97	DYRK4	CMGC		98	94	89	87	
98	EEF2K	ATYPICAL		102	89	95	94	
99	EGFR	TK		100	99	105	93	
100	EIF2AK2	OTHER		97	113	94	114	
101	EIF2AK3	OTHER		94	102	93	92	
102	EPHA1	TK		83	71	91	85	
103	EPHA2	TK		116	94	105	107	
104	EPHA3	TK		96	86	92	94	
105	EPHA4	TK		97	88	86	90	

**106**	**EPHA5**	**TK**		96	67	93	60	
**107**	**EPHA6**	**TK**		110	80	100	94	
**108**	**EPHA7**	**TK**		103	101	85	96	
**109**	**EPHA8**	**TK**		98	91	110	91	
**110**	**EPHB1**	**TK**		113	103	113	95	
**111**	**EPHB2**	**TK**		93	86	86	84	
**112**	**EPHB3**	**TK**		100	69	100	85	
**113**	**EPHB4**	**TK**		99	74	100	82	
**114**	**ERBB2**	**TK**		96	79	99	86	
**115**	**ERBB4**	**TK**		97	71	98	81	
**116**	**ERK1**	**CMGC**		99	99	100	102	
**117**	**ERK2**	**CMGC**		101	90	95	93	
**118**	**ERK5**	**CMGC**		80	103	90	93	
**119**	**ERK7**	**CMGC**		96	85	99	98	
**120**	**FAK**	**TK**		116	88	104	88	
**121**	**FER**	**TK**		101	94	110	90	
**122**	**FES**	**TK**		81	67	79	90	
**123**	**FGFR1**	**TK**		93	78	93	79	
**124**	**FGFR2**	**TK**		90	51	97	74	
**125**	**FGFR3**	**TK**		98	76	110	95	
**126**	**FGFR4**	**TK**		96	67	90	79	
**127**	**FGR**	**TK**		108	82	105	84	
**128**	**FLT3**	**TK**		101	94	98	91	
**129**	**FRK**	**TK**		107	104	107	102	
**130**	**FYN**	**TK**		90	55	104	83	
**131**	**GRK2**	**AGC**		97	97	84	88	
**132**	**GRK3**	**AGC**		118	144	122	138	
**133**	**GRK4**	**AGC**		116	86	103	130	
**134**	**GRK5**	**AGC**		104	106	102	103	
**135**	**GRK6**	**AGC**		117	114	113	121	
**136**	**GRK7**	**AGC**		121	123	108	116	
**137**	**GSG2**	**OTHER**		92	78	93	68	
**138**	**GSK3alpha**	**CMGC**		109	90	112	106	
**139**	**GSK3beta**	**CMGC**		88	88	82	90	
**140**	**HCK**	**TK**		107	95	99	76	
**141**	**HIPK1**	**CMGC**		100	91	90	93	
**142**	**HIPK2**	**CMGC**		97	101	90	95	
**143**	**HIPK3**	**CMGC**		88	87	89	90	
**144**	**HIPK4**	**CMGC**		92	84	95	91	
**145**	**HRI**	**OTHER**		120	123	98	129	
**146**	**IGF1R**	**TK**		97	101	104	80	
**147**	**IKKalpha**	**OTHER**		102	102	104	93	
**148**	**IKKbeta**	**OTHER**		110	99	84	79	
**149**	**IKKepsilon**	**OTHER**		103	101	95	92	
**150**	**INSR**	**TK**		95	92	95	84	
**151**	**INSRR**	**TK**		92	90	92	83	
**152**	**IRAK1**	**TKL**		107	106	100	104	
**153**	**IRAK4**	**TKL**		97	100	92	93	
**154**	**ITK**	**TK**		94	75	93	82	
**155**	**JAK1**	**TK**		88	85	77	88	
**156**	**JAK2**	**TK**		94	85	90	89	
**157**	**JAK3**	**TK**		80	75	83	79	
**158**	**JNK1**	**CMGC**		100	76	90	81	
**159**	**JNK2**	**CMGC**		87	79	88	97	
**160**	**JNK3**	**CMGC**		96	119	83	92	
**161**	**KIT**	**TK**		87	78	88	78	
**162**	**LCK**	**TK**		107	66	102	84	
**163**	**LIMK1**	**TKL**		102	87	89	96	
**164**	**LIMK2**	**TKL**		95	82	98	93	
**165**	**LRRK2**	**TKL**		89	92	94	90	
**166**	**LTK**	**TK**		115	50	95	77	
**167**	**LYN**	**TK**		96	95	104	88	
**168**	**MAP3K1**	**STE**		96	83	96	93	
**169**	**MAP3K10**	**STE**		106	84	96	91	
**170**	**MAP3K11**	**STE**		109	118	108	110	
**171**	**MAP3K7/MAP3K7IP1**	**STE**		88	91	91	91	
**172**	**MAP3K9**	**STE**		102	96	89	103	
**173**	**MAP4K2**	**STE**		94	71	95	93	
**174**	**MAP4K4**	**STE**		89	95	94	85	
**175**	**MAP4K5**	**STE**		98	94	92	92	
**176**	**MAPKAPK2**	**CAMK**		121	77	101	78	
**177**	**MAPKAPK3**	**CAMK**		109	50	98	65	
**178**	**MAPKAPK5**	**CAMK**		127	89	101	97	
**179**	**MARK1**	**CAMK**		91	90	87	99	
**180**	**MARK2**	**CAMK**		103	104	107	99	
**181**	**MARK3**	**CAMK**		106	103	102	105	
**182**	**MARK4**	**CAMK**		99	94	102	91	
**183**	**MASTL**	**AGC**		99	92	94	99	
**184**	**MATK**	**TK**		101	86	94	95	
**185**	**MEK1**	**STE**		112	114	109	109	
**186**	**MEK2**	**STE**		102	88	100	89	
**187**	**MEK5**	**STE**		118	114	104	94	
**188**	**MEKK2**	**STE**		108	108	99	101	
**189**	**MEKK3**	**STE**		95	104	94	102	
**190**	**MELK**	**CAMK**		93	90	107	90	
**191**	**MERTK**	**TK**		103	73	101	85	
**192**	**MET**	**TK**		101	89	95	91	
**193**	**MINK1**	**STE**		89	73	86	57	
**194**	**MKK4**	**STE**		94	110	96	111	
**195**	**MKK6 SDTD**	**STE**		89	109	85	137	
**196**	**MKK7**	**STE**		101	110	84	90	
**197**	**MKNK1**	**CAMK**		90	95	92	91	
**198**	**MKNK2**	**CAMK**		112	115	104	107	
**199**	**MLK4**	**TKL**		94	102	99	99	
**200**	**MST1**	**STE**		102	92	103	98	
**201**	**MST2**	**STE**		94	82	90	75	
**202**	**MST3**	**STE**		122	114	106	103	
**203**	**MST4**	**STE**		90	82	92	80	
**204**	**MTOR**	**ATYPICAL**		93	92	94	94	
**205**	**MUSK**	**TK**		98	93	97	83	
**206**	**MYLK**	**CAMK**		92	78	81	64	
**207**	**MYLK2**	**CAMK**		93	82	85	79	
**208**	**MYLK3**	**CAMK**		97	70	103	80	
**209**	**NEK1**	**OTHER**		98	96	96	93	
**210**	**NEK11**	**OTHER**		97	110	86	104	
**211**	**NEK2**	**OTHER**		105	97	96	101	
**212**	**NEK3**	**OTHER**		104	92	96	97	
**213**	**NEK4**	**OTHER**		102	81	98	94	
**214**	**NEK6**	**OTHER**		95	87	96	92	
**215**	**NEK7**	**OTHER**		109	118	108	121	
**216**	**NEK9**	**OTHER**		86	93	96	81	
**217**	**NIK**	**STE**		93	103	103	103	
**218**	**NLK**	**CMGC**		96	96	97	91	
**219**	**p38alpha**	**CMGC**		78	73	17	4	
**220**	**p38beta**	**CMGC**		71	50	3	2	
**221**	**p38delta**	**CMGC**		93	85	91	98	
**222**	**p38gamma**	**CMGC**		93	84	88	52	
**223**	**PAK1**	**STE**		87	88	84	80	
**224**	**PAK2**	**STE**		101	68	91	69	
**225**	**PAK3**	**STE**		88	74	92	79	
**226**	**PAK4**	**STE**		97	92	98	82	
**227**	**PAK6**	**STE**		101	98	99	90	
**228**	**PAK7**	**STE**		99	92	101	93	

**229**	**PASK**	**CAMK**		107	97	95	97		
**230**	**PBK**	**OTHER**		128	93	95	106		
**231**	**PDGFRalpha**	**TK**		107	95	96	92		
**232**	**PDGFRbeta**	**TK**		100	99	98	99		
**233**	**PDK1**	**AGC**		102	57	98	80		
**234**	**PHKG1**	**CAMK**		95	75	96	86		
**235**	**PHKG2**	**CAMK**		96	93	102	95		
**236**	**PIM1**	**CAMK**		88	74	96	70		
**237**	**PIM2**	**CAMK**		109	92	100	86		
**238**	**PIM3**	**CAMK**		90	89	89	102		
**239**	**PKA**	**AGC**		97	21	86	49		
**240**	**PKCalpha**	**AGC**		95	82	92	73		
**241**	**PKCbeta1**	**AGC**		91	92	93	87		
**242**	**PKCbeta2**	**AGC**		105	84	97	90		
**243**	**PKCdelta**	**AGC**		98	82	107	66		
**244**	**PKCepsilon**	**AGC**		107	107	104	103		
**245**	**PKCeta**	**AGC**		116	115	116	110		
**246**	**PKCgamma**	**AGC**		77	78	81	63		
**247**	**PKCiota**	**AGC**		94	71	82	77		
**248**	**PKCmu**	**AGC**		106	94	95	96		
**249**	**PKCnu**	**AGC**		88	65	88	69		
**250**	**PKCtheta**	**AGC**		104	82	106	84		
**251**	**PKCzeta**	**AGC**		92	102	87	96		
**252**	**PKMYT1**	**OTHER**		102	93	98	95		
**253**	**PKMzeta**	**AGC**		105	98	103	88		
**254**	**PKN3**	**AGC**		104	101	91	92		
**255**	**PLK1**	**OTHER**		103	90	94	96		
**256**	**PLK3**	**OTHER**		115	114	104	104		
**257**	**PRK1**	**AGC**		112	101	94	101		
**258**	**PRK2**	**AGC**		94	97	90	96		
**259**	**PRKD2**	**CAMK**		87	92	82	81		
**260**	**PRKG1**	**AGC**		120	96	113	90		
**261**	**PRKG2**	**AGC**		94	83	88	84		
**262**	**PRKX**	**AGC**		102	88	95	85		
**263**	**PYK2**	**TK**		108	79	87	80		
**264**	**RAF1 YDYD**	**TKL**		14	3	36	12		
**265**	**RET**	**TK**		91	70	95	89		
**266**	**RIPK2**	**TKL**		104	89	99	84		
**267**	**RIPK4**	**TKL**		103	98	103	100		
**268**	**RIPK5**	**TKL**		100	99	101	100		
**269**	**ROCK1**	**AGC**		99	92	99	102		
**270**	**ROCK2**	**AGC**		101	102	103	98		
**271**	**RON**	**TK**		104	77	89	83		
**272**	**ROS**	**TK**		99	91	97	85		
**273**	**RPS6KA1**	**AGC**		110	96	96	95		
**274**	**RPS6KA2**	**AGC**		95	84	95	75		
**275**	**RPS6KA3**	**AGC**		97	80	90	84		
**276**	**RPS6KA4**	**AGC**		93	93	95	95		
**277**	**RPS6KA5**	**AGC**		104	97	96	93		
**278**	**RPS6KA6**	**AGC**		90	83	88	81		
**279**	**S6K**	**AGC**		102	92	88	91		
**280**	**S6Kbeta**	**AGC**		96	85	102	96		
**281**	**SAK**	**OTHER**		92	81	89	71		
**282**	**SGK1**	**AGC**		108	85	99	97		
**283**	**SGK2**	**AGC**		101	93	97	96		
**284**	**SGK3**	**AGC**		103	98	96	97		
**285**	**SIK1**	**CAMK**		84	86	89	83		
**286**	**SIK2**	**CAMK**		89	81	93	82		
**287**	**SIK3**	**CAMK**		95	87	96	99		
**288**	**SLK**	**STE**		98	93	94	91		
**289**	**SNARK**	**CAMK**		107	92	105	98		
**290**	**SNK**	**OTHER**		102	90	94	92		
**291**	**SRC**	**TK**		117	105	107	100		
**292**	**SRMS**	**TK**		102	93	98	97		
**293**	**SRPK1**	**CMGC**		94	71	96	68		
**294**	**SRPK2**	**CMGC**		100	94	111	102		
**295**	**STK17A**	**CAMK**		111	76	105	88		
**296**	**STK23**	**CAMK**		89	79	78	76		
**297**	**STK25**	**STE**		100	89	90	91		
**298**	**STK33**	**CAMK**		109	96	93	99		
**299**	**STK39**	**STE**		108	106	90	100		
**300**	**SYK**	**TK**		105	89	95	94		
**301**	**TAOK2**	**STE**		90	97	104	108		
**302**	**TAOK3**	**STE**		105	98	99	91		
**303**	**TBK1**	**OTHER**		103	101	93	93		
**304**	**TEC**	**TK**		88	69	94	80		
**305**	**TGFBR1**	**TKL**		95	88	88	91		
**306**	**TGFBR2**	**TKL**		79	74	82	79		
**307**	**TIE2**	**TK**		96	66	87	78		
**308**	**TLK1**	**AGC**		125	131	103	114		
**309**	**TLK2**	**AGC**		135	162	120	207		
**310**	**TNK1**	**TK**		101	72	89	85		
**311**	**TRKA**	**TK**		107	94	101	97		
**312**	**TRKB**	**TK**		105	74	101	92		
**313**	**TRKC**	**TK**		109	89	112	97		
**314**	**TSF1**	**OTHER**		107	88	97	94		
**315**	**TSK2**	**CAMK**		97	93	88	99		
**316**	**TSSK1**	**CAMK**		112	105	102	102		
**317**	**TTBK1**	**CK1**		108	126	101	115		
**318**	**TTBK2**	**CK1**		109	126	105	113		
**319**	**TTK**	**OTHER**		99	95	91	89		
**320**	**TXK**	**TK**		100	90	97	97		
**321**	**TYK2**	**TK**		95	83	102	93		
**322**	**TYRO3**	**TK**		96	77	104	92		
**323**	**ULK2**	**OTHER**		113	103	99	113		
**324**	**VEGFR1**	**TK**		100	93	101	93		
**325**	**VEGFR2**	**TK**		98	77	84	79		
**326**	**VEGFR3**	**TK**		108	84	105	88		
**327**	**VRK1**	**CK1**		102	103	100	130		
**328**	**VRK2**	**CK1**		113	114	107	108		
**329**	**WEE1**	**OTHER**		103	100	100	95		
**330**	**WNK1**	**OTHER**		99	97	93	94		
**331**	**WNK2**	**OTHER**		95	100	90	96		
**332**	**WNK3**	**OTHER**		107	109	93	104		
**333**	**YES**	**TK**		87	70	98	77		
**334**	**ZAK**	**TKL**		109	102	106	106		
**335**	**ZAP70**	**TK**		118	103	114	91		
**Selectivity Score (< 50 % residual activity):**				**0.003**	**0.015**	**0.012**	**0.024**	**#DIV/0!**	**#DIV/0!**

***Classification of protein kinase families (Manning et al. Science 6 December 2002: Vol. 298 no. 5600 pp. 1912-1934):**
AGC: containing PKA, PKG and PKC families
CAMK: Calcium/Calmoduline-dependent protein kinases
CK1: Casein kinase 1 -like
CMGC: containing CDK, MAPK ,GSK3 and CLK families
TK: Tyrosine Kinase
TKL: Tyrosine Kinase-like
STE: Homologs of Yeast Sterile 7, Sterile 11, Sterile 20 Kinases

MKK6 SDTD, RAF1 YDYD: Constitutively active variants
