# Supplementary material for: Target Hopping from Protein Kinases to PXR: Identification of Small-Molecule Protein Kinase Inhibitors as Selective Modulators of Pregnane X Receptor from TüKIC Library
Source: Cells. 2022 Apr 12;11(8):1299. doi: 10.3390/cells11081299 (PMC9030254; doi:10.3390/cells11081299)
Supplement: Supplementary file 1 [file cells-11-01299-s001.zip › Supplementary_Information.pdf]

Supplementary Information

Target hopping from protein kinases to PXR: identification of small-molecule protein kinase inhibitors as selective modulators of pregnane X receptor from TüKIC library

Enni-Kaisa Mustonen (1), Tatu Pantsar (2,3), Azam Rashidian (4), Juliander Reiner (2), Matthias Schwab (1,5,6), Stefan Laufer (2,6,7), Oliver Burk (1)

(1) Dr. Margarete Fischer-Bosch-Institute of Clinical Pharmacology, Stuttgart, and University of Tübingen, Tübingen, Germany

(2) Department of Pharmaceutical and Medicinal Chemistry, Institute of Pharmaceutical Sciences, University of Tübingen, Tübingen, Germany

(3) School of Pharmacy, Faculty of Health Sciences, University of Eastern Finland, Kuopio, Finland

(4) Department of Internal Medicine VIII, University Hospital Tübingen, Tübingen, Germany

(5) Departments of Clinical Pharmacology and Biochemistry and Pharmacy, University of Tuebingen, Tübingen, Germany

(6) Cluster of Excellence iFIT (EXC 2180) "Image-Guided and Functionally Instructed Tumor Therapies", University of Tübingen, Tübingen, Germany

(7) Tuebingen Center for Academic Drug Discovery & Development (TüCAD2), 72076, Tübingen, Germany

Content

Supplementary methods and compound NMR spectra……..…………………......…………….2

Supplementary figures…………………………………………………………………..15

Supplementary tables……………………………………………………………………22

Supplementary Methods: Compound synthesis

General information

Reagents and solvents were purchased from Sigma-Aldrich, VWR international, Merck KGaA, Alfa Aesar, Acros, Roth, Fisher Scientific, Fluka, OxChem, FluoroChem, TCI or abcr and used without further purification. LRMS were determined by DC-MS (Advion expressionS CMS, ESITLC analyses were performed on fluorescent TLC Silica gel 60 F254 aluminum sheets (Merck KGaA) with UV illumination (254/366 nm). The puriflash 430 automated flash chromatography system in combination with standard glass or plastics columns were used for (flash-)chromatography with Davisil LC60A 20-45 micron silica from Grace Davison or Geduran Si60 63-200 micron silica from Merck KGaA as stationary phase. The used mobile phases are indicated in the experimental section. Purity of all compounds was determined via reverse phase HPLC on Hewlett Packard HP 1090 Series II LC equipped with a UV diode array detector (DAD, detection at 230 nm and 254 nm) and was ≥95% for tested compounds. The chromatographic separation was performed on a Phenomenex Luna 5u C8 column (150 mm x 4.6 mm, 5 µm) at 35 °C oven temperature with an injection volume of 5 µL (gradient: 0.01 M KH_2PO_4, pH 2.3 (Solvent A), methanol (Solvent B): 40 % B to 85 % B in 8 min, 85 % B for 5 min, 85% to 40 % B in 1 min, 40 % B for 2 min, flow: 1.5 mL/min, total time 16 min). NMR spectra were measured with a Bruker Avance 200 or Bruker Avance 400 and analyzed with MestReNova v6.0.2-5475 (Mestrelab Research S.L). Residual solvent peaks were used for calibration: 1H-spectra: 2.55 ppm (DMSO-d_6), 7.27 ppm ($CDCl_3$). 13C-spectra: 39.51 ppm (DMSO-d_6), 77.00 ppm ($CDCl_3$). Chemical shifts (δ) are reported in parts per million (ppm), the solvent used is indicated in the experimental section.

5-(3-Chlorophenyl)-pent-4-enoic acid (1)

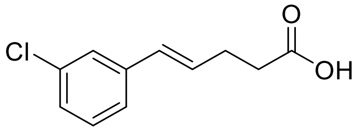

8,7 mL of a sodium methanolate solution (30 % wt in methanol 46,5 mmol; 2 eq.) were added dropwise to a stirred suspension of 10 g (3-carboxypropyl)triphenylphosphonium bromide (23,29 mmol; 1 eq.) 10 mL dry methanol at rt. After that, the reaction mixture was heated under reflux for further 30 min, before 4,9 g 3-chlorebenzaldehyde (35,94 mmol, 1,5 eq.) were added dropwise and heating to reflux continued overnight. Upon consumption of the starting material, the resulting reddish suspension was allowed to cool to room temperature, poured in 100 mL water, strongly acidified with conc. Aqueous hydrochloric acid, and extracted several times with EtOAc. The combined organic phases were dried over sodium sulfate and the volatiles removed *in vacuo*. The pure product was obtained by flash chromatography (EtOAc/hexanes/HCOOH 85/15+1%) as yellowish oil (3,74 g, 76 %)

HPLC: 7,59 min

ESI-MS: *m/z* for $C_{11}H_{11}ClO_2$ $[M–H]^–$ = 208,8; M = 210,66

Melting point: liquid at rt

^{1}H-NMR: (200 MHz, DMSO) δ = 12.34 (s, 1H), 7.47 – 7.14 (m, 4H), 6.47 – 6.31 (m, 2H), 2.46 – 2.31 (m, 4H). ppm

5-(3-Chlorophenyl)-pentanoic acid (2)

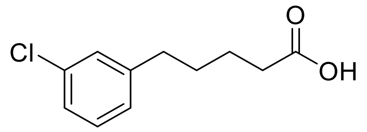

4,14 g **1** (19,66 mmol; 1 eq.) were dissolved in 20 mL ethyl acetate, 0,208 g palladium on activated coal (10 % wt 0,17 mmol; 0,01 eq.) was added and the resulting suspension thoroughly stirred under hydrogen atmosphere upon complete conversion (TLC-control). The reaction mixture was filtrated over celite and the volatiles removed *in vacuo.* The product was obtained as pale yellow oil and used without further purifcation. (3,81 g, 91 %)

HPLC:	6,65 min
ESI-MS:	*m/z* for $C_{11}H_{13}ClO_2$ $[M–H]^–$ = 210,8; M = 212,67
Melting point:	liquid at rt
IR:	2943, 2855, 1699, 1573, 1403, 1253, 1194, 1078, 911, 778, 699, 541 cm^{-1}
^{1}H-NMR:	(200 MHz, DMSO) δ = 12.26 (s, 1H), 7.39 – 7.09 (m, 4H), 2.58 (t, *J* = 7.1 Hz, 2H), 2.22 (t, *J* = 6.9 Hz, 2H), 1.67 – 1.30 (m, 4H) ppm.

2-Chloro-6,7,8,9-tetrahydro-5*H*-benzo[7]annulen-5-on (3)

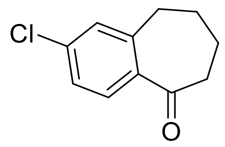

6,16 g **2** (28,97 mmol; 1 eq.) and a few drops DMF were dissolved in 30 mL DCM and 4,41 g oxalyl chloride (34,77 mmol; 1,2 eq.) added dropwise whilst stirring at room temperature. Upon completion of the activation indicated by ceasing of gas evolution, the reaction mixture was cooled to 0 °C, 11,53 g aluminium-(III)-chloride (86,92 mmol; 3 eq.) were added in portions and the suspension stirred for another 20-30 minutes. The reaction was quenched by pouring on a mixture of ice and 6N aqueous hydrochloric acid and extracted several times with DCM. The combined organic layers were washed with 5 % aqueous sodium hydroxide solution, dried over sodium sulfate and the volatiles removed *in vacuo.* The product was obtained as pale yellow oil (3,69 g, 60 %) after flash-chromatography (hexanes/DCM 80/20->40/60 within 1 h)

HPLC:	7,51 min (90,65 %)
ESI-MS:	*m/z* for $C_{11}H_{11}ClO$ n. d.; M = 194,66
Melting point:	liquid at rt
IR:	2938, 2855, 1727, 1677, 1590, 1452, 1282, 1261, 1215, 1086, 961, 816, 782, 749 cm^{-1}
^{1}H-NMR:	(200 MHz, DMSO) δ = 7.59 (d, *J* = 8.0 Hz, 1H), 7.44 – 7.34 (m, 2H), 2.99 – 2.80 (m, 2H), 2.75 – 2.55 (m, 2H), 1.71 (dq, *J* = 22.7, 6.3 Hz, 4H) ppm.
^{13}C-NMR:	(50 MHz, DMSO) δ = 203.85 (s), 143.69 (s), 137.04 (s), 136.76 (s), 130.07 (s), 129.47 (s), 126.57 (s), 40.12 (s), 31.07 (s), 24.48 (s), 20.12 (s) ppm.

N-Benzyl-3-nitrobenzamide (4a)

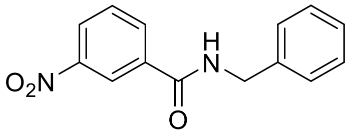

200 mg 3-nitrobenzoic acid (1,2 mmol, 1 eq.) and 213 mg CDI (1,32 mmol; 1,1 eq) were dissolved in 5 mL dry THF and stirred until the gas evolution ceased. Following, 128 mg benzylamine (1,2 mmol; 1 eq.) was added and the mixture was stirred at rt until total consumption of the starting material (TLC-control), poured in water and extracted several times with DCM. The combined organic phases were dried over sodium sulfate, filtered, and the volatiles removed *in vacuo*. The remaining off-white solid was used without further purification. (297 mg, 97 %)

HPLC:	6,05 min (97,86 %)
ESI-MS:	*m/z* for $C_{13}H_{12}N_2O_3$ $[M+Na]^+$ = 279,0; $[M+Na+MeOH]^+$ = 311,1; $[M–H]^–$ = 254,9 M = 256,26
Melting point:	51,9 °C
IR:	3296, 3084, 3029, 1639, 1523, 1444, 1348, 1310, 1048, 932, 836, 740, 657, 611 cm^{-1}

General procedure for preparing 3-nitroanilides

To a stirred solution of the carboxylic acid and catalytic amounts of DMF was dropwise added oxalyl chloride or thionyl chloride and stirring continued at rt until the gas evolution ceased and TLC indicated no remaining starting material. Alternatively, a commercially available carboxylic acid chloride was used. After that, a solution of the respective aniline in THF and triethylamine (4 mmol, 1 eq.) were added and continued in stirring until TLC indicated no further conversion. The reaction mixture was poured in water and extracted several times with EtOAc. The combined organic phases were dried over sodium sulfate, filtered, and the volatiles removed *in vacuo.* The remaining was washed with ice cold ether to obtain the pure title compounds.

N-(4-Methyl-3-nitro-phenyl)-2-phenyl-acetamide (4b)

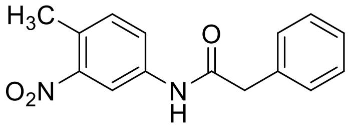

The title compound was synthesized according to the general procedure above from 750 mg phenylacetic acid (5,51 mmol, 1 eq.), 1,05 g oxalyl chloride (8,26 mmol; 1,5 eq.), 838 mg 4-methyl-3-nitroanilin (5,51 mmol; 1 eq.) and 557mg triethylamine (5,51 mmol; 1eq.) The title compound was obtained as yellowish solid (64 %).

HPLC:	6,89 min (99,55 %)
ESI-MS:	*m/z* for $C_{15}H_{14}N_2O_3$ $[M+Na]^+$ = 293,1; $[M–H]^–$ = 268,9; M = 270,29
Melting point:	125,2 °C
IR:	3234, 315, 3109, 3046, 2972, 2926, 1656, 1606, 1515, 1336, 1286, 1136, 891, 824, 728, 512

^{1}H-NMR: (200 MHz, DMSO-*d6*) δ = 10.53 (s, 1H), 8.36 (d, *J* = 2.1 Hz, 1H), 7.72 (dd, *J* = 8.3, 2.2 Hz, 1H), 7.40 (d, *J* = 8.4 Hz, 1H), 7.32 – 7.08 (m, 5H), 3.65 (s, 2H), 2.43 (s, 3H) ppm.

N-(4-Fluoro-3-nitro-phenyl)-2-phenyl-acetamid (4c)

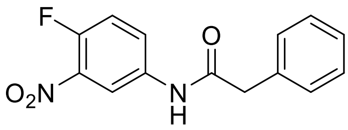

The title compound was synthesized according to the general procedure above from 500 mg phenylacetic acid (3,67 mmol; 1 eq.), 436 mg thionyl chloride (3,67 mg; 1 eq.), 573 mg 4-fluoro-3-nitroanilin (3,67 mmol; 1 eq.) and 371 mg triethylamine (3,67 mmol; 1 eq.). The title compound was obtained as yellowish solid (80 %).

HPLC: 6,57 min (98,65 %)

ESI-MS: *m/z* for $C_{14}H_{11}FN_2O_3$ $[M+Na]^+$ = 297,1; $[M–H]^-$ = 272,9; M = 274,25

Melting point: 131,5 °C

IR: 3242, 3192, 3138, 3071, 1652, 1606, 1532, 1494, 1403, 1340, 1249, 1140, 886, 828, 762, 724 cm^{-1}

^{1}H-NMR: (200 MHz, DMSO-*d6*) δ = 10.63 (s, 1H), 8.51 (dd, *J* = 6.9, 2.7 Hz, 1H), 7.86 (ddd, *J* = 9.0, 3.9, 2.9 Hz, 1H), 7.52 (dd, *J* = 11.1, 9.1 Hz, 1H), 7.38 – 7.16 (m, 5H), 3.66 (s, 2H) ppm.

N-(4-Fluoro-3-nitro-phenyl)-3-fluoro-benzamide (4d)

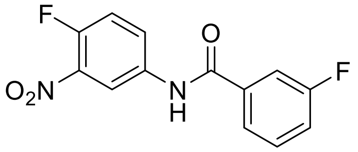

The title compound was synthesized according to the general procedure above from 500 mg 3-fluorobenzoeic acid (3,57 mmol; 1 eq.), 467 mg thionyl chloride (3,93mmol; 1,1 eq.), 557 mg 4-fluoro-3-nitroanilin (3,57 mmol; 1 eq.) and 361 mg triethylamine (3,57 mmol; 1 eq.). The title compound was obtained as yellowish solid (54 %).

Yield: 534,4 mg = 53,8 %

HPLC: 7,29 min (89,22 %)

ESI-MS: *m/z* for $C_{13}H_8F_2N_2O_3$ $[M–H]^-$ = 276,9; M = 278,21

Melting point: 166,0 °C

IR: 3321, 3300, 3067, 1656, 1527, 1340, 1219, 886, 841, 803, 749, 512 cm^{-1}

^{1}H-NMR: (200 MHz, DMSO-*d6*) δ = 10.74 (s, 1H), 8.69 (dd, *J* = 6.9, 2.6 Hz, 1H), 8.22 – 8.06 (m, 1H), 7.82 (m, 2H), 7.69 – 7.37 (m, 3H) ppm.

Reduction of nitroarenes

The respective nitroaryl was dissolved in ethyl acetate, palladium on activated coal (10 % wt, 0,01-0,03 eq.) was added and the resulting suspension thoroughly stirred under hydrogen atmosphere upon complete conversion (TLC-control). The reaction mixture was filtrated over celite and the volatiles removed *in vacuo.* The yielded solid was, depending on its purity, whether used without further purification or purified as indicated.

N-Benzyl-3-aminobenzamid (5a)

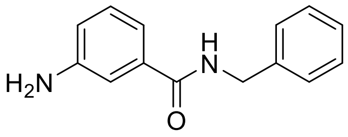

The title compound was synthesized according to the general procedure above from 690 mg **4a** (2,69 mmol) and obtained as white solid after washing with hexanes/ether (9/1) (93 %)

HPLC:	3,29 min (99,36 %)
ESI-MS:	*m/z* for $C_{14}H_{14}N_2O$ $[M+Na]^+$ = 249,0; $[M–H]^–$ = 224,9; M = 226,28
Melting point:	95,9 °C
IR:	3438, 3342, 3288, 3026, 1631, 1573, 1536, 1486, 2323, 1265, 990, 870, 687, 504 cm^{-1}
^{1}H-NMR:	(200 MHz, DMSO-*d6*) δ = 8.81 (t, *J* = 5.9 Hz, 1H), 7.40 – 7.18 (m, 5H), 7.15 – 6.97 (m, 3H), 6.76 – 6.64 (m, 1H), 5.25 (s, 2H), 4.44 (d, *J* = 6.0 Hz, 2H) ppm.

N-(2-Methyl-5-amino-phenyl)-2-phenyl-acetamide (5b)

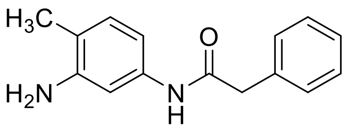

The title compound was synthesized according to the general procedure above from 870 mg **4b** (3,22 mmol) and obtained as beige solid after washing with ice cold ether (89 %)

HPLC:	3,41 min (100 %)
ESI-MS:	*m/z* for $C_{15}H_{16}N_2O$ $[M+Na]^+$ = 263,1; $[M–H]^–$ = 238,9; M = 240,31
Melting point:	150,3 °C
IR:	3396, 3288, 3055, 3026, 2963, 1673, 1602, 1540, 1511, 1444, 1415, 1344, 1319, 874, 816, 707, 553, 487 cm^{-1}
^{1}H-NMR:	(200 MHz, DMSO-*d6*) δ = 9.78 (s, 1H), 7.37 – 7.14 (m, 5H), 6.93 (d, *J* = 1.8 Hz, 1H), 6.79 (d, *J* = 8.0 Hz, 1H), 6.66 (dd, *J* = 8.0, 2.0 Hz, 1H), 4.80 (s, 2H), 3.35 (s, 2H), 1.98 (s, 3H) ppm.

N-(3-Amino-4-fluorophenyl)-2-phenyl-acetamide (5c)

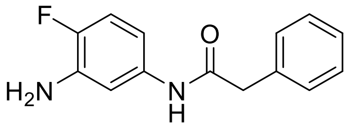

The title compound was synthesized according to the general procedure above from 760 mg **4c** (2,77 mmol) and obtained as brown solid after washing with ice cold ether (33 %)

HPLC:	4,33 min (98,40 %)
ESI-MS:	*m/z* for $C_{14}H_{13}FN_2O$ $[M+Na]^+$ = 267,1; $[M–H]^–$ = 242,9; M = 244,27
Melting point:	188,0 °C
IR:	3392, 3275, 3250, 3059, 1669, 1619, 1556, 1507, 1436, 1240, 1194, 866, 807, 762, 699, 562 cm^{-1}
^{1}H-NMR:	(200 MHz, DMSO-*d6*) δ = 9.89 (s, 1H), 7.37 – 7.20 (m, 5H), 7.10 (dd, *J* = 8.4, 1.9 Hz, 1H), 6.86 (dd, *J* = 10.9, 9.2 Hz, 1H), 6.72 – 6.61 (m, 1H), 5.12 (s, 2H), 3.57 (s, 2H) ppm.

N-(3-Amino-4-fluoro-phenyl)-3-fluor-benzamide (5d)

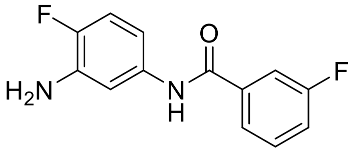

The title compound was synthesized according to the general procedure above from 497 mg **4d** (1,79 mmol) and obtained as ochre solid after purification by flash chromatography (hexanes/EtOAc 70/30) (35 %)

HPLC:	4,99 min
ESI-MS:	*m/z* for $C_{13}H_{10}F_2N_2O$ $[M+Na]^+$ = 271,0; $[M+Na+MeOH]^+$ = 303,0; M = 248,23
	Neg. Mode: *m/z* $[M–H]^–$ = 246,9 (ber.: 247,1)
Melting point:	103,2 °C
^{1}H-NMR:	(200 MHz, DMSO-*d6*) δ = 10.08 (s, 1H), 7.92 – 7.66 (m, 2H), 7.67 – 7.48 (m, 1H), 7.42 (td, *J* = 8.7, 2.5 Hz, 1H), 7.29 (dd, *J* = 8.5, 2.3 Hz, 1H), 7.05 – 6.92 (m, 1H), 6.91 – 6.72 (m, 1H), 5.19 (s, 2H) ppm.

Buchwald-Hartwig-crosscoupling

An argon flushed round bottom flask was charged with chlorobenzosuberone (1,00-1,05 eq), respective arylamine (1,0 eq.) , caesium carbonate (3 eq.), XPhos (0,5 eq.) and palladium-(II)-acetate (0,1 eq.) and equipped with a reflux condeonsor. The mixture of starting materials was suspended in 10 mL of a mixture of 1,4-dioxane and tert-butanol (4/1) and heated to reflux for about 30 minutes whilst stirring. After consumption of the arylamine (TLC control), the reaction mixture was poured in ammonium chloride solution (aq., sat.) and

extracted three times with EtOAc. The combined organic phases were dried over sodium sulfate and the volatiles removed *in vacuo*. The remaining solid was purified by flash chromatography as indicated.

N-((Phenyl)-methyl)-3-[(5-oxo-5*H*-6,7,8,9-tetrahydro-benzo[7]annulen-2-yl)amino]-benzamide (12)

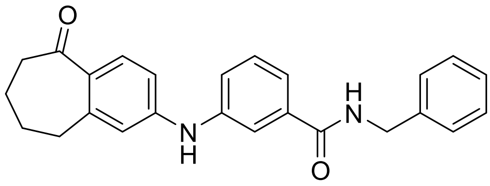

According to the general procedure above **compound 12** was synthesized from 81 mg **3** (0,42 mmol; 1,05 eq.), 90 mg **5a** (0,40 mmol; 1 eq.), 95 mg XPhos (0,20 mmol; 0,25 eq.), 9 mg palladium(II)acetate (0,04 mmol; 0,1 eq.) and 390 mg cesium carbonate (1,19 mmol; 3 eq.) and obtained after flash-chromatography (DCM/EA/nHEX 30:15:55 to 30:60:10 within 1,5h) as light yellow solid (56 %).

HPLC: 7,81 min (96,37 %)

ESI-MS: *m/z* for $C_{25}H_{24}N_2O_2$ $[M+Na]^+$ = 407,1; $[M–H]^–$ = 383,0; M = 384,48

Melting point: 168,4 °C

IR: 3408, 3283, 3059, 2930, 2859, 1647, 1573, 1535, 1481, 1314, 1094, 873, 732, 690, 594, 511 cm^{-1}

^{1}H-NMR: (400 MHz, DMSO-*d6*) δ = 9.03 (t, *J* = 6.0 Hz, 1H), 8.82 (s, 1H), 7.72 – 7.67 (m, 1H), 7.60 (d, *J* = 8.5 Hz, 1H), 7.50 – 7.45 (m, 1H), 7.40 (t, *J* = 7.8 Hz, 1H), 7.36 – 7.29 (m, 5H), 7.27 – 7.21 (m, 1H), 6.97 (dd, *J* = 8.6, 2.3 Hz, 1H), 6.88 (d, *J* = 2.2 Hz, 1H), 4.47 (d, *J* = 6.0 Hz, 2H), 2.90 – 2.80 (m, 2H), 2.68 – 2.59 (m, 2H), 1.85 – 1.73 (m, 2H), 1.73 – 1.63 (m, 2H) ppm.

^{13}C-NMR: (50 MHz, DMSO-*d6*) δ = 201.91 (s), 166.15 (s), 147.41 (s), 144.21 (s), 141.76 (s), 139.68 (s), 135.64 (s), 130.62 (s), 129.28 (s), 129.14 (s), 128.24 (s, 2C), 127.14 (s, 2C), 126.70 (s), 121.46 (s), 120.05 (s), 117.84 (s), 115.45 (s), 112.76 (s), 42.59 (s), 40.19 (s), 32.18 (s), 24.63 (s), 20.23 (s) ppm.

N-(((3-((5-Oxo-6,7,8,9-tetrahydro-5*H*-benzo[7]annulen-2-yl)-amino)-4-methyl)-phenyl)-2-phenyl-acetamide (73)

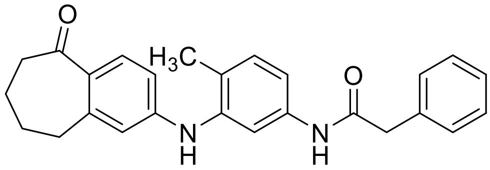

According to the general procedure above **compound 73** was synthesized from 100 mg **3** (0,51 mmol; 1 eq.), 123 mg **5b** (0,51 mmol; 1 eq.), 122 mg XPhos (0,26 mmol; 0,5 eq.), 12 mg palladium(II)acetate (0,05 mmol; 0,1 eq.) und 502 mg cesium carbonate (1,54 mmol; 3eq.) and obtained after flash chromatography (DCM/hexanes/EtOAc 3:5:2) as yellow solid (45 %).

HPLC: 9,59 min (96,94 %)

ESI-MS: *m/z* for $C_{26}H_{26}N_2O_2$ $[M+Na]^+$ = 421,1; $[M–H]^–$ = 397,0; M = 398,51

Melting point: 93,8 °C

IR: 3292, 2926, 2855, 1652, 1581, 1519, 1490, 1340, 1319, 1273, 1107, 820, 695, 449 cm^{-1}

^{1}H-NMR: (400 MHz, DMSO-*d6*) δ = 10.10 (s, 1H), 8.05 (s, 1H), 7.59 (s, 1H), 7.55 (d, *J* = 8.5 Hz, 1H), 7.36 – 7.28 (m, 4H), 7.25 (d, *J* = 7.5 Hz, 2H), 7.15 (d, *J* = 8.2 Hz, 1H), 6.72 – 6.66 (m, 1H), 6.65 – 6.61 (m, 1H), 3.60 (s, 2H), 2.86 – 2.75 (m, 2H), 2.64 – 2.58 (m, 2H), 2.13 (s, 3H), 1.81 – 1.71 (m, 2H), 1.71 – 1.59 (m, 2H) ppm.

^{13}C-NMR: (50 MHz, DMSO-*d6*) δ = 201.68 (s), 168.88 (s), 149.34 (s), 144.17 (s), 139.35 (s), 137.76 (s), 136.00 (s), 130.98 (s), 130.58 (s), 129.05 (s, 2C), 128.25 (s, 2C), 127.92 (s), 126.47 (s), 126.25 (s), 114.83 (s), 114.55 (s), 113.71 (s), 111.87 (s), 43.31 (s), 40.19 (s), 32.28 (s), 24.59 (s), 20.24 (s), 17.31 (s) ppm.

N-(((3-((5-Oxo-6,7,8,9-tetrahydro-5*H*-benzo[7]annulen-2-yl)-amino)-4-fluoro)-phenyl)-2-phenyl-acetamide (100)

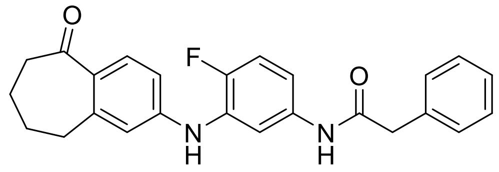

According to the general procedure above **compound 100** was synthesized from 100 mg **3** (0,51 mmol; 1 eq.), 125 mg **5c** (0,51 mmol; 1 eq.), 122 mg XPhos (0,26 mmol; 0,5 eq.), 12 mg palladium(II)acetat (0,05 mmol; 0,1eq.) and 502 mg cesium carbonate (1,54 mmol; 3eq.) and obtained after flash-chromatography (DCM/hexanes/EtOAc 3:5:2) as light brown solid (116 mg, 56 %).

HPLC: 7,61 min (95,73 %)

ESI-MS: *m/z* for $C_{25}H_{23}FN_2O_2$ $[M+Na]^+$ = 425,1; $[M–H]^–$ = 401,0; M = 402,47

Melting point: 65,1 °C

IR: 3280, 2926, 2855, 1652, 1577, 1490, 1344, 1244, 1203, 1107, 803, 687, 445 cm^{-1}

^{1}H-NMR: (400 MHz, DMSO-*d6*) δ = 10.19 (s, 1H), 8.47 (s, 1H), 7.73 (dd, *J* = 7.6, 2.2 Hz, 1H), 7.57 (d, *J* = 8.5 Hz, 1H), 7.35 – 7.29 (m, 4H), 7.28 – 7.22 (m, 2H), 7.22 – 7.15 (m, 1H), 6.82 (dd, *J* = 8.6, 1.4 Hz, 1H), 6.77 – 6.73 (m, 1H), 3.62 (s, 2H),

2.89 – 2.77 (m, 2H), 2.68 – 2.56 (m, 2H), 1.82 – 1.72 (m, 2H), 1.71 – 1.62 (m, 2H). ppm.

^{13}C-NMR: (101 MHz, DMSO) δ = 202.01 (s), 169.02 (s), 150.49 (d, *J* = 240.7 Hz), 147.72 (s), 143.96 (s), 135.87 (s), 135.84 (d, *J* = 2.5 Hz), 130.36 (s), 129.05 (s), 128.79 (d, *J* = 12.4 Hz), 128.29 (s), 126.53 (s), 116.09 (d, *J* = 20.4 Hz), 115.45 (s), 114.18 (d, *J* = 7.1 Hz), 113.25 (s), 112.69 (s), 43.27 (s), 40.19 (s), 32.19 (s), 24.61 (s), 20.21 (s).ppm.

N-(((3-((5-Oxo-6,7,8,9-tetrahydro-5*H*-benzo[7]annulen-2-yl)-amino)-4-fluoro)-phenyl)-3-fluorobenzamide (109)

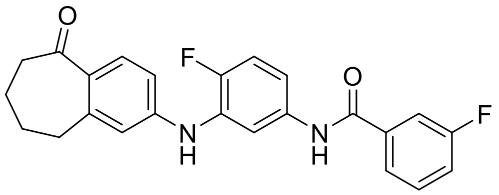

According to the general procedure above **compound 109** was synthesized from 74 mg **3** (0,38 mmol; 1,05 eq.), 90 mg **5d** (0,36 mmol; 1 eq.), 86 mg XPhos (0,18 mmol; 0,5 eq.), 8 mg Palladium(II)acetat (0,04 mmol; 0,1 eq.) und 364 mg cesium carbonate (1,10 mmol; 3 eq.) and obtained after flash chromatography (DCM/hexanes/EtOAc 30:55:15 to 30:10:60 within 1,5h) as light yellow solid (70 %).

HPLC: 8,16 min (98,74 %)

ESI-MS: *m/z* for $C_{24}H_{20}F_2N_2O_2$ $[M+Na]^+$ = 429,1; $[M–H]^–$ = 405,0; M = 406,43

Melting point: 91,5 °C

IR: 3296, 2934, 2859, 1644, 1590, 1523, 1436, 1469, 1249, 1095, 791, 741, 449 cm^{-1}

^{1}H-NMR: (200 MHz, DMSO-*d6*) *δ* = 10.33 (s, 1H), 8.53 (s, 1H), 7.91 (dd, *J* = 7.7, 2.5 Hz, 1H), 7.82 – 7.77 (m, 1H), 7.78 – 7.72 (m, 1H), 7.63 – 7.55 (m, 2H), 7.50 – 7.41 (m, 2H), 7.27 (dd, *J* = 10.8, 8.9 Hz, 1H), 6.88 (dd, *J* = 8.5, 1.9 Hz, 1H), 6.82 (d, *J* = 2.0 Hz, 1H), 2.90 – 2.79 (m, 2H), 2.69 – 2.58 (m, 2H), 1.83 – 1.73 (m, 2H), 1.73 – 1.64 (m, 2H) ppm.

^{13}C-NMR: (50 MHz, DMSO-*d6*) *δ* = 202.06 (s), 164.10 (d, *J* = 2.5 Hz), 161.91 (d, *J* = 244.4 Hz), 150.87 (d, *J* = 241.4 Hz), 147.70 (s), 144.01 (s), 137.11 (d, *J* = 6.8 Hz), 135.51 (d, *J* = 2.6 Hz), 130.61 (d, *J* = 8.1 Hz), 130.41 (s), 129.11 (s), 128.80 (d, *J* = 12.6 Hz), 123.88 (d, *J* = 2.8 Hz), 118.52 (d, *J* = 21.2 Hz), 116.05 (d, *J* = 20.6 Hz), 115.51 (s), 115.40 (d, *J* = 7.1 Hz), 114.47 (d, *J* = 22.9 Hz), 114.40 (s), 112.73 (s), 40.21 (s), 32.20 (s), 24.63 (s), 20.24 (s) ppm

Experimental ^{1}H- and ^{13}C-NMR spectra of compounds 12, 73, 100 and 109

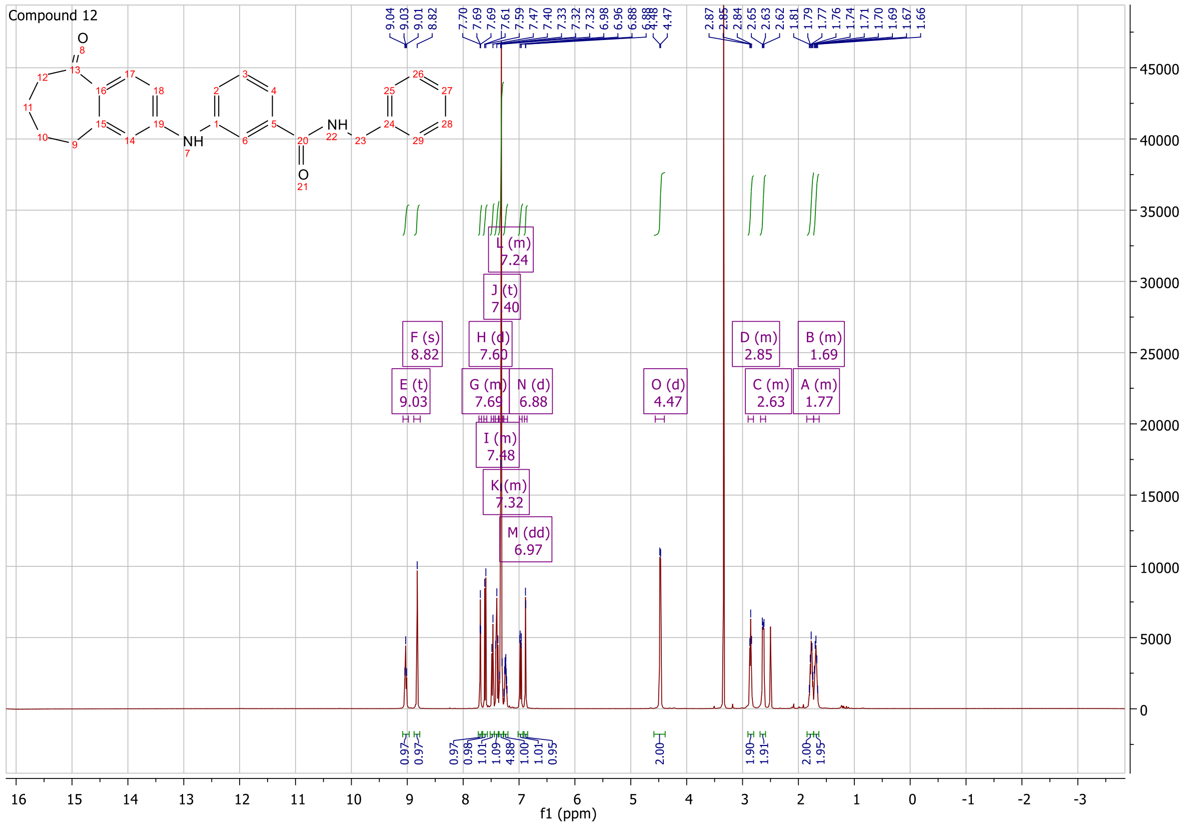

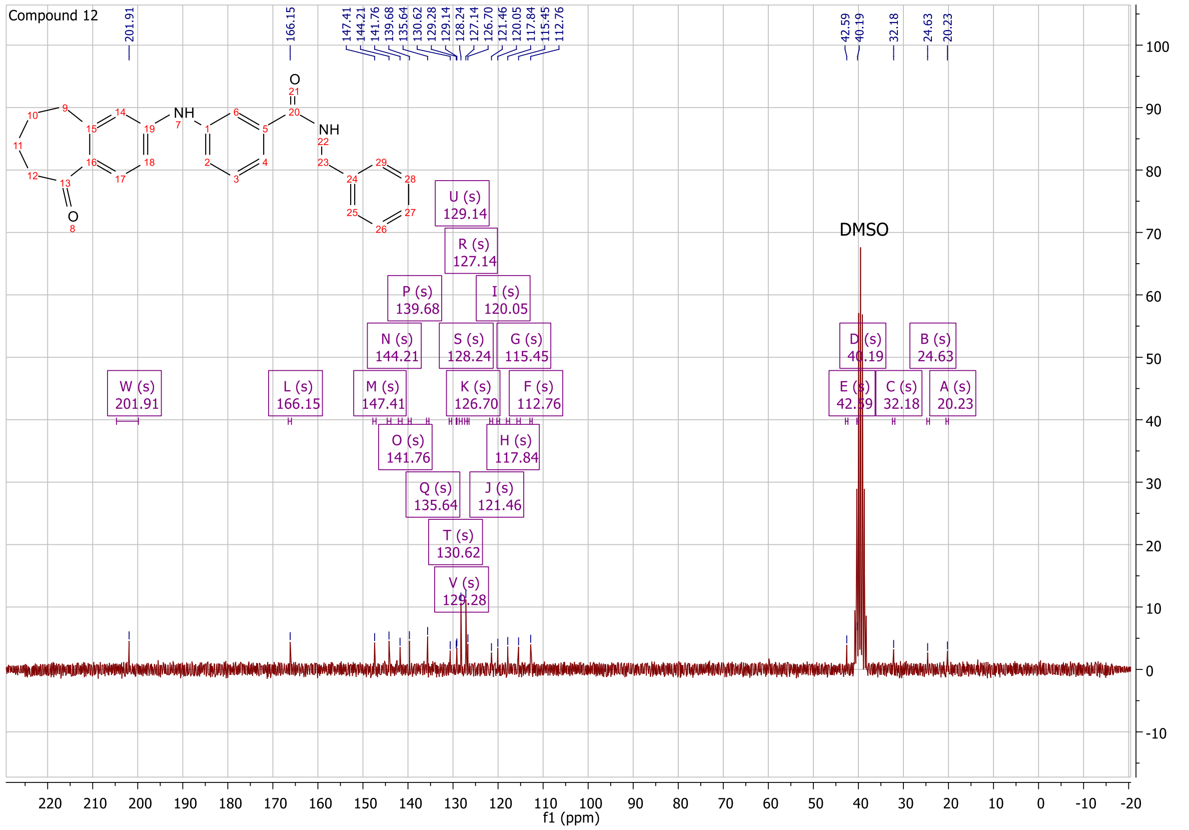

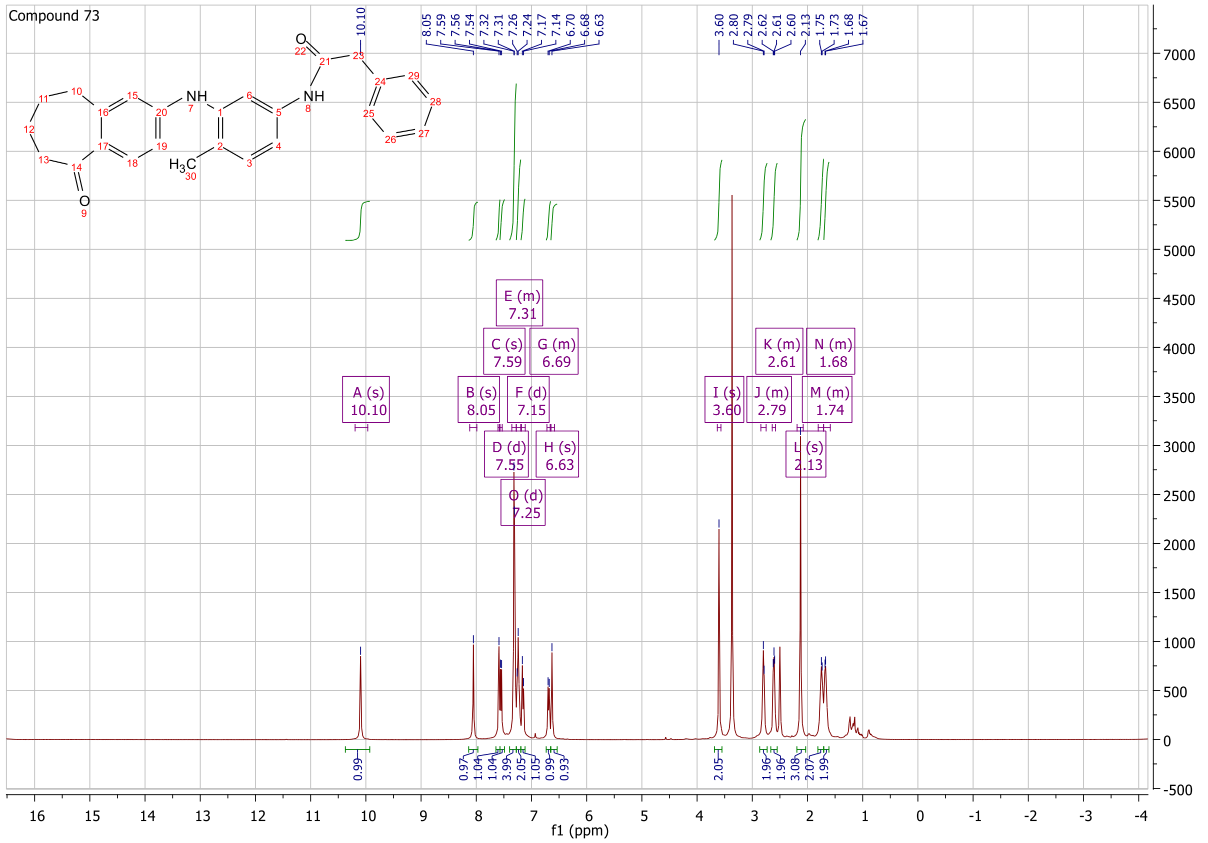
Compound 73
10.10
8.05 7.59 7.56 7.54 7.32 7.31 7.26 7.24 7.17 7.14 6.70 6.68 6.63
3.60 2.80 2.79 2.62 2.61 2.60 2.13 1.75 1.73 1.68 1.67
A (s) 10.10
B (s) 8.05
C (s) 7.59
D (d) 7.55
E (m) 7.31
F (d) 7.15
G (m) 6.69
H (s) 6.63
I (s) 3.60
J (m) 2.79
K (m) 2.61
L (s) 2.13
M (m) 1.74
N (m) 1.68
O (d) 7.25
0.99
0.97 1.04 1.04 3.99 2.05 1.05 0.99 0.93
2.05
1.96 1.96 3.08 2.07 1.99
f1 (ppm)

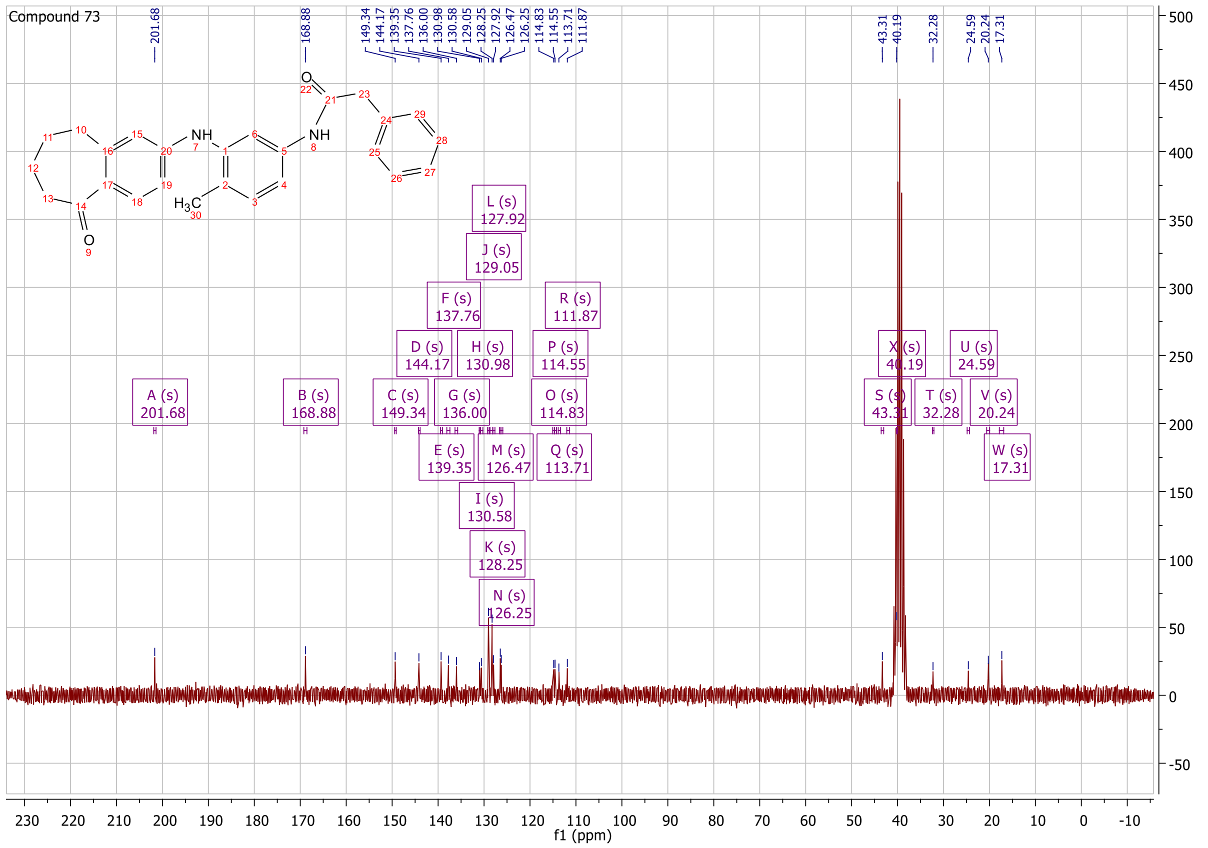
Compound 73
201.68
168.88
149.34 144.17 139.35 137.76 136.00 130.98 130.58 129.05 128.25 127.92 126.47 126.25 114.83 114.55 113.71 111.87
43.31 40.19 32.28 24.59 20.24 17.31
A (s) 201.68
B (s) 168.88
C (s) 149.34
D (s) 144.17
E (s) 139.35
F (s) 137.76
G (s) 136.00
H (s) 130.98
I (s) 130.58
J (s) 129.05
K (s) 128.25
L (s) 127.92
M (s) 126.47
N (s) 126.25
O (s) 114.83
P (s) 114.55
Q (s) 113.71
R (s) 111.87
S (s) 43.31
T (s) 32.28
U (s) 24.59
V (s) 20.24
W (s) 17.31
X (s) 40.19
f1 (ppm)

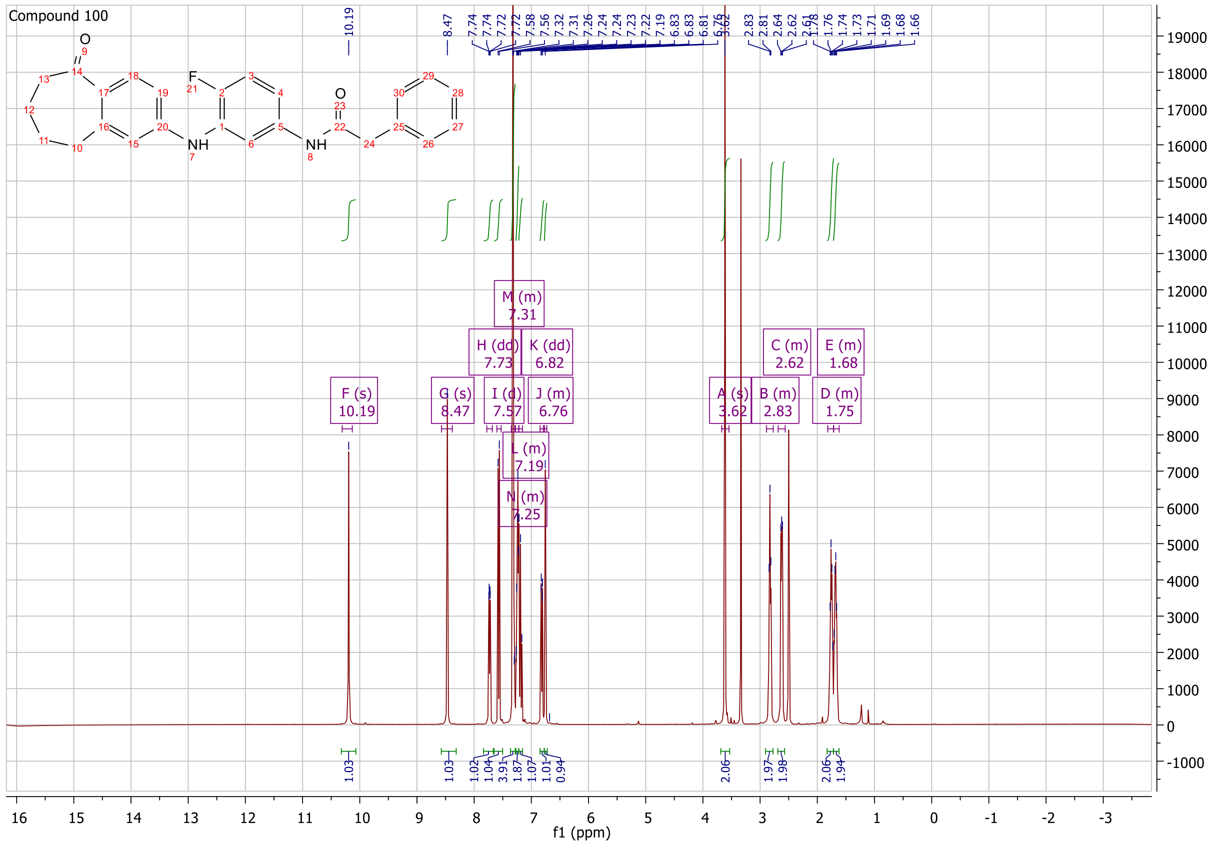
Compound 100
f1 (ppm)

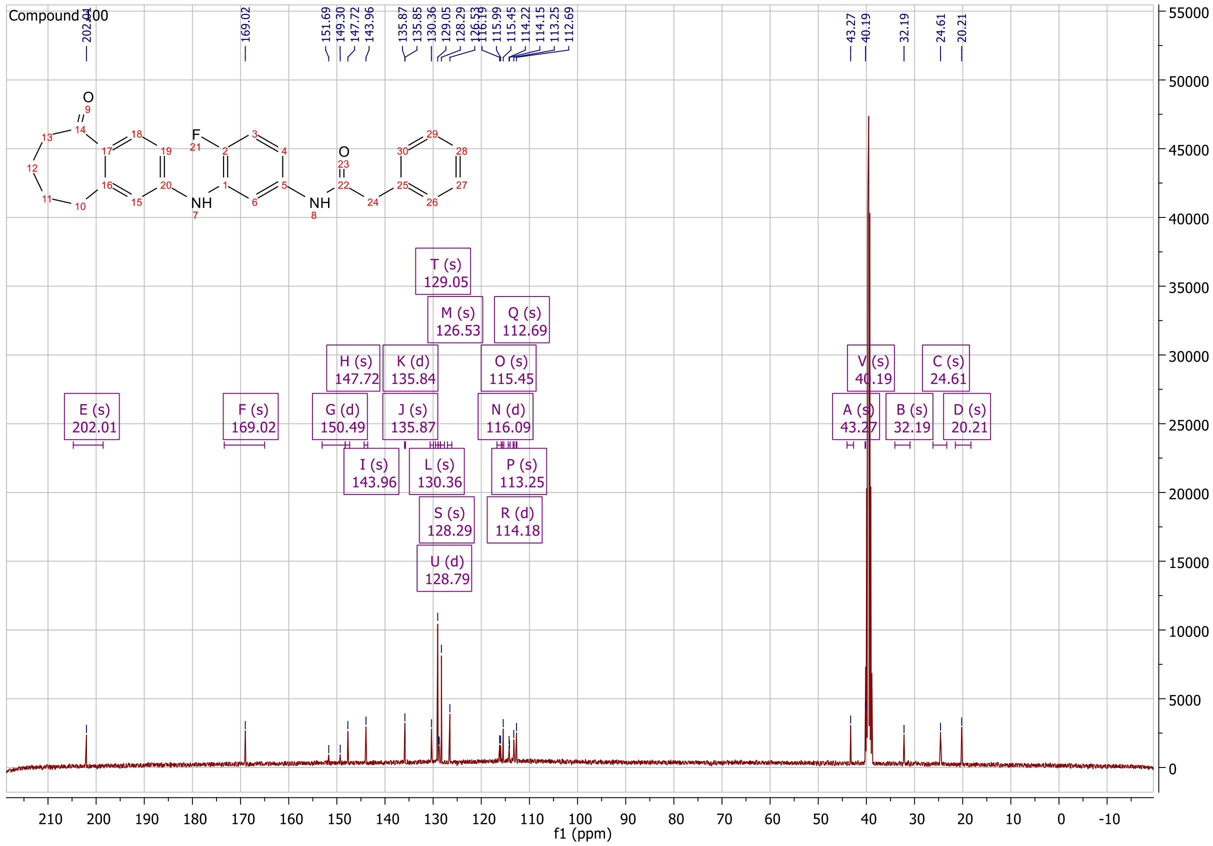
Compound 100
f1 (ppm)

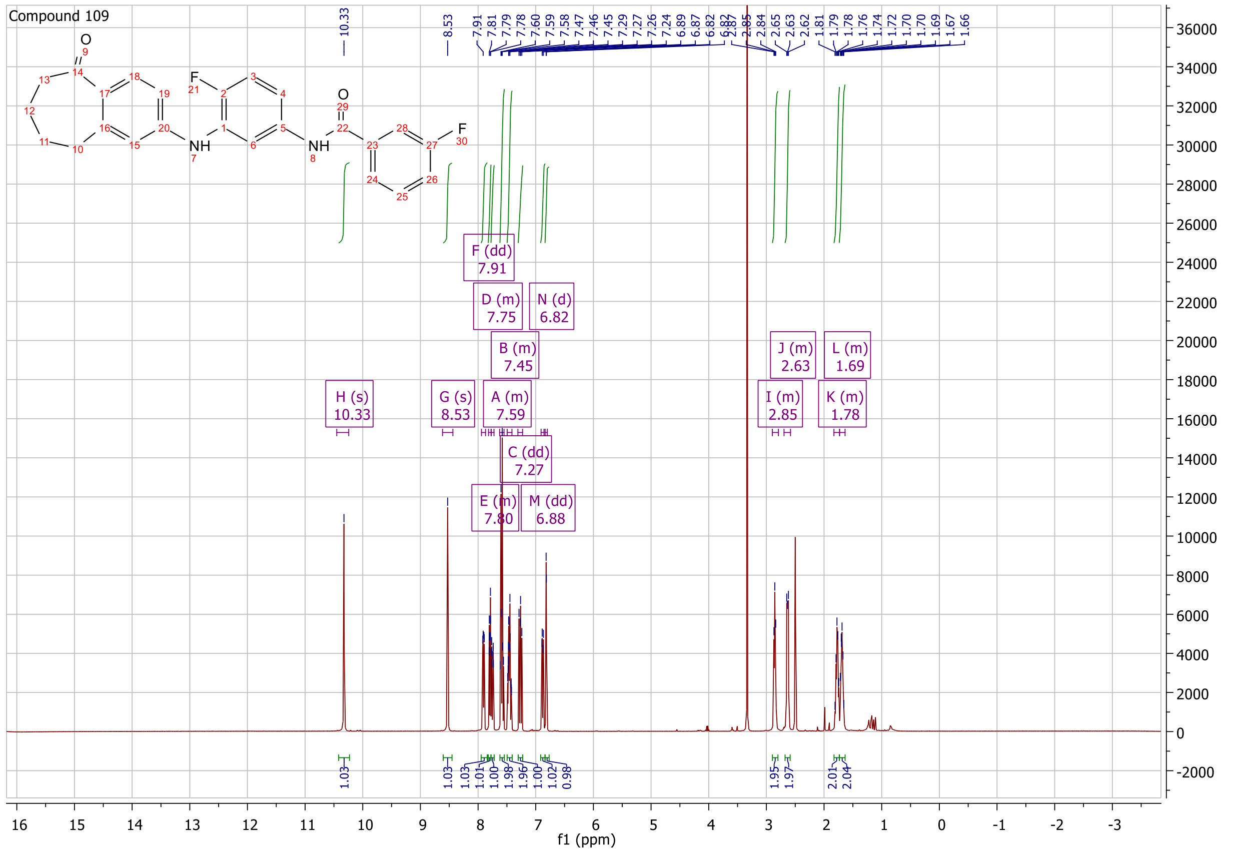
Compound 109
f1 (ppm)

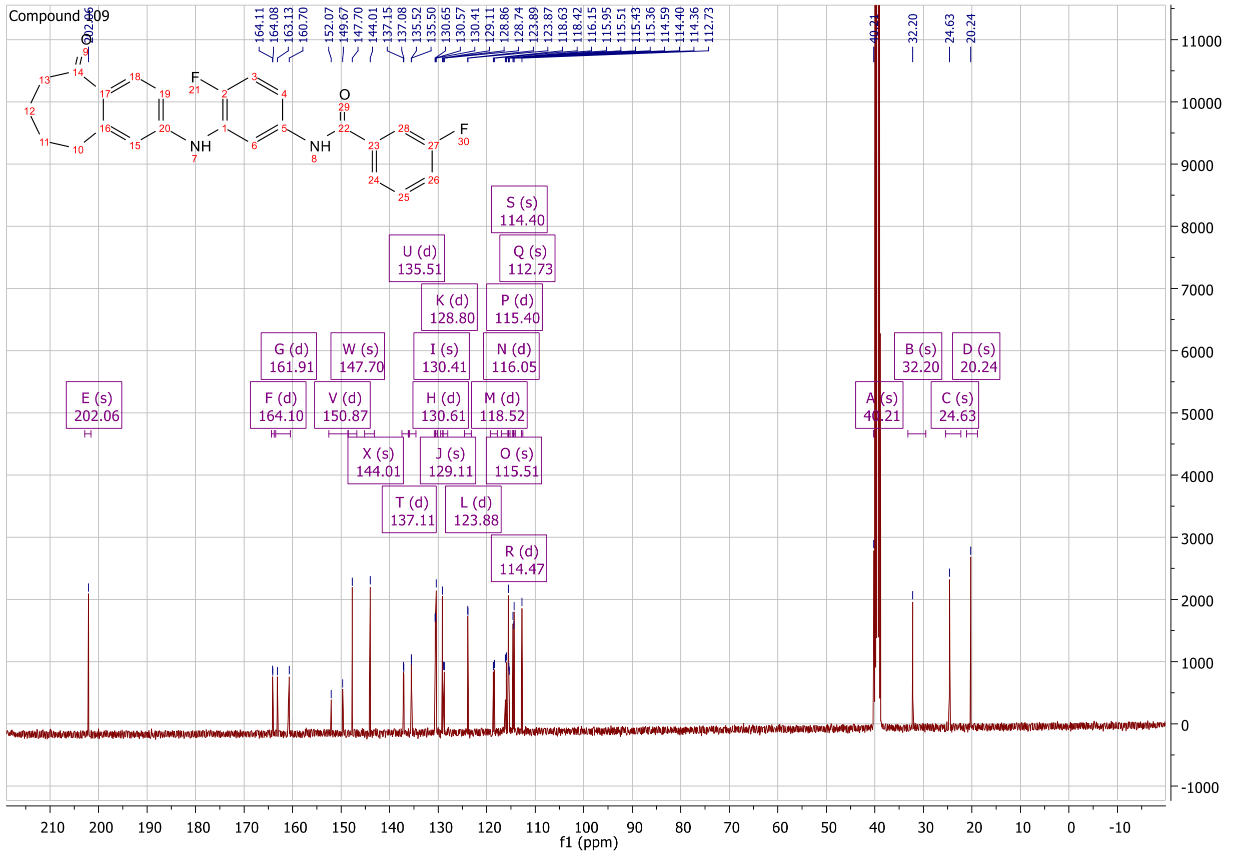
Compound 109
f1 (ppm)

Supplementary Figures

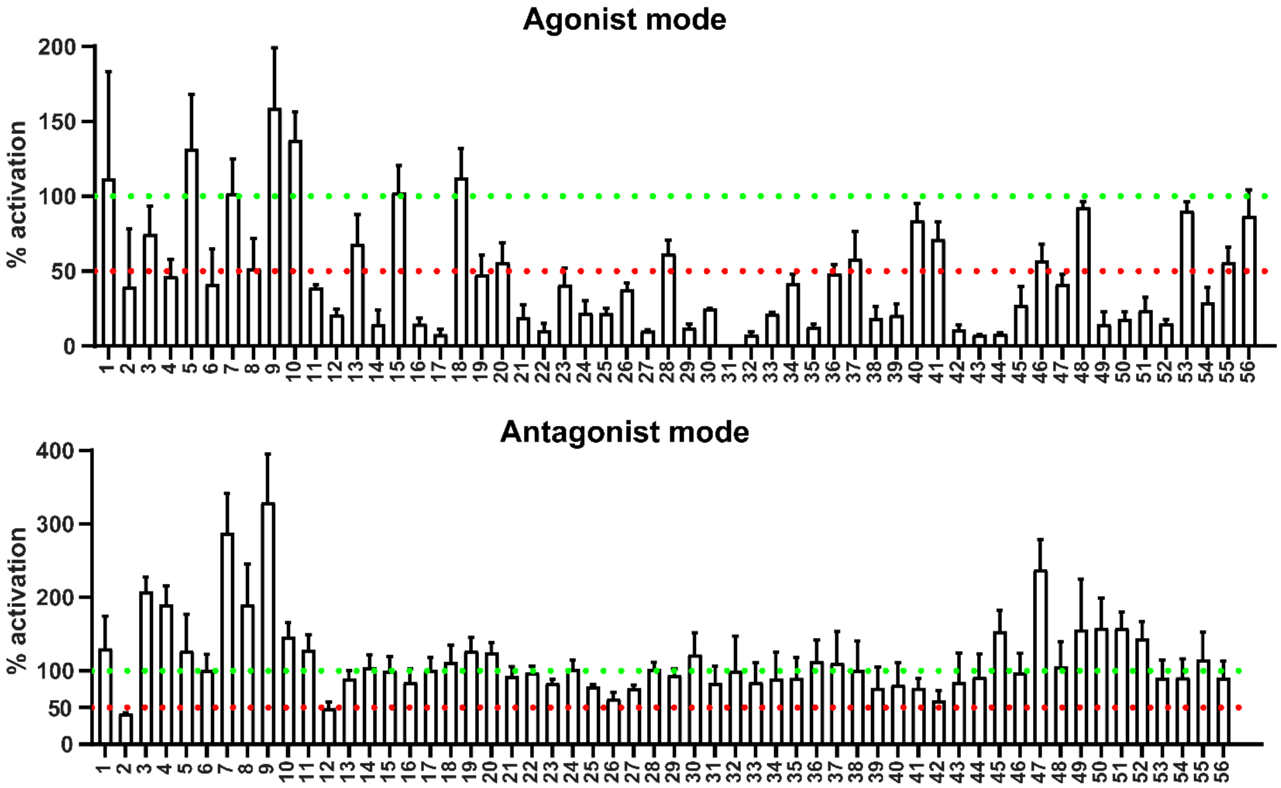

Supplementary Figure S1. Effects of 56 in silico screened TüKIC compounds (A) alone or (B) in combination with 10 μM rifampicin on PXR-mediated transactivation of CYP3A4 reporter gene. H-P cells were transiently transfected with CYP3A4 reporter gene, treated with 0.1% (A) or 0.2% (B) DMSO, 10 μM rifampicin, 10 μM test compounds or co-treated with 10 μM rifampicin and 10 μM test compounds. Luciferase activities were measured after 24 h treatment. Data is expressed as mean ± SD %. Activation was calculated according to Zhu et al. [38] from three independent experiments with technical triplicates. Fold induction achieved by 10 μM rifampicin was set as 100%. Green and red dashed lines represent the 100% and 50% activation, respectively.

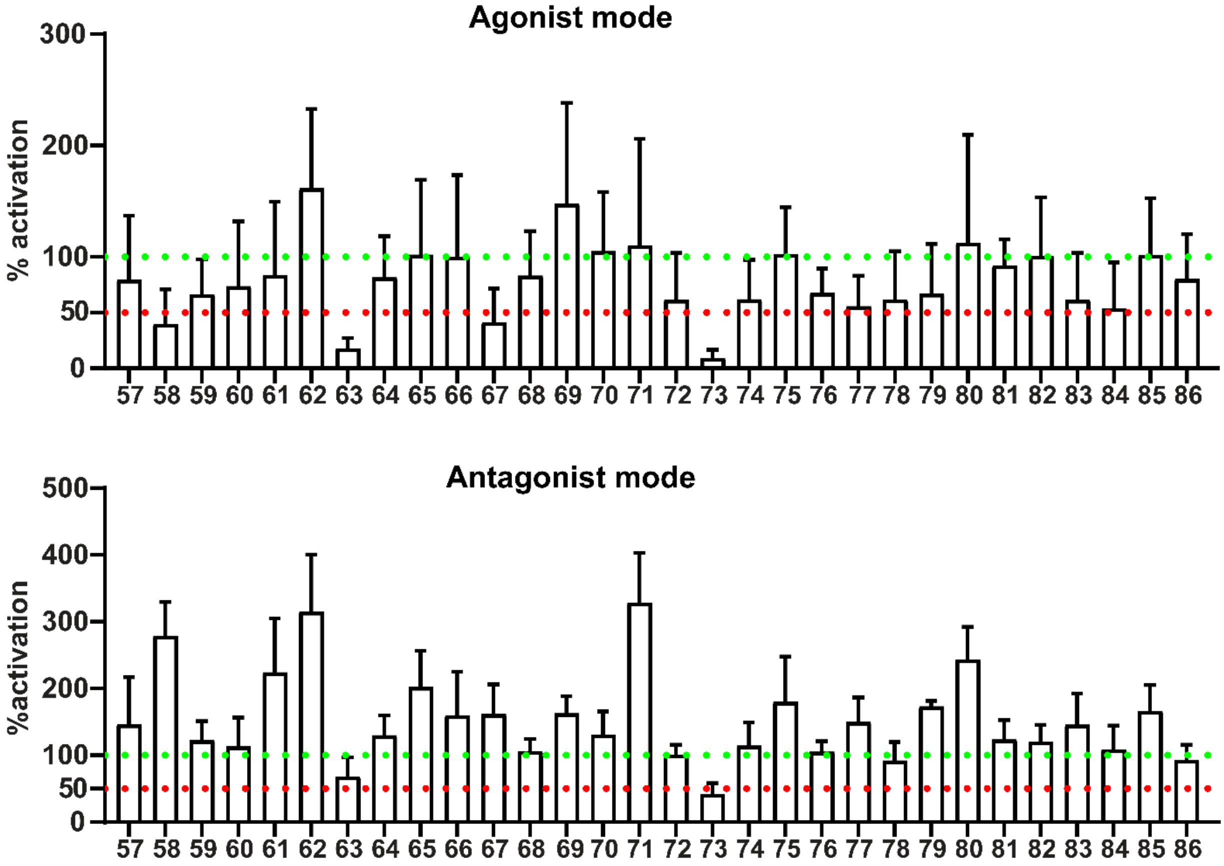

Supplementary Figure S2. Effects of first round structural analogues (A) alone or (B) in combination with rifampicin on PXR-mediated transactivation of CYP3A4 reporter gene. H-P cells were transiently transfected with CYP3A4 reporter gene, treated with 0.1% (A) or 0.2% (B) DMSO, 10 µM rifampicin, 10 µM of test compounds or co-treated with 10 µM rifampicin and 10 µM test compounds. Luciferase activities were measured after 24 h treatment. Data is expressed as mean ± SD %. Activation was calculated according to Zhu et al. [38] from three independent experiments with technical triplicates. Fold induction achieved by 10 µM rifampicin was set as 100%. Green and red dashed lines represent the 100% and 50% activation, respectively.

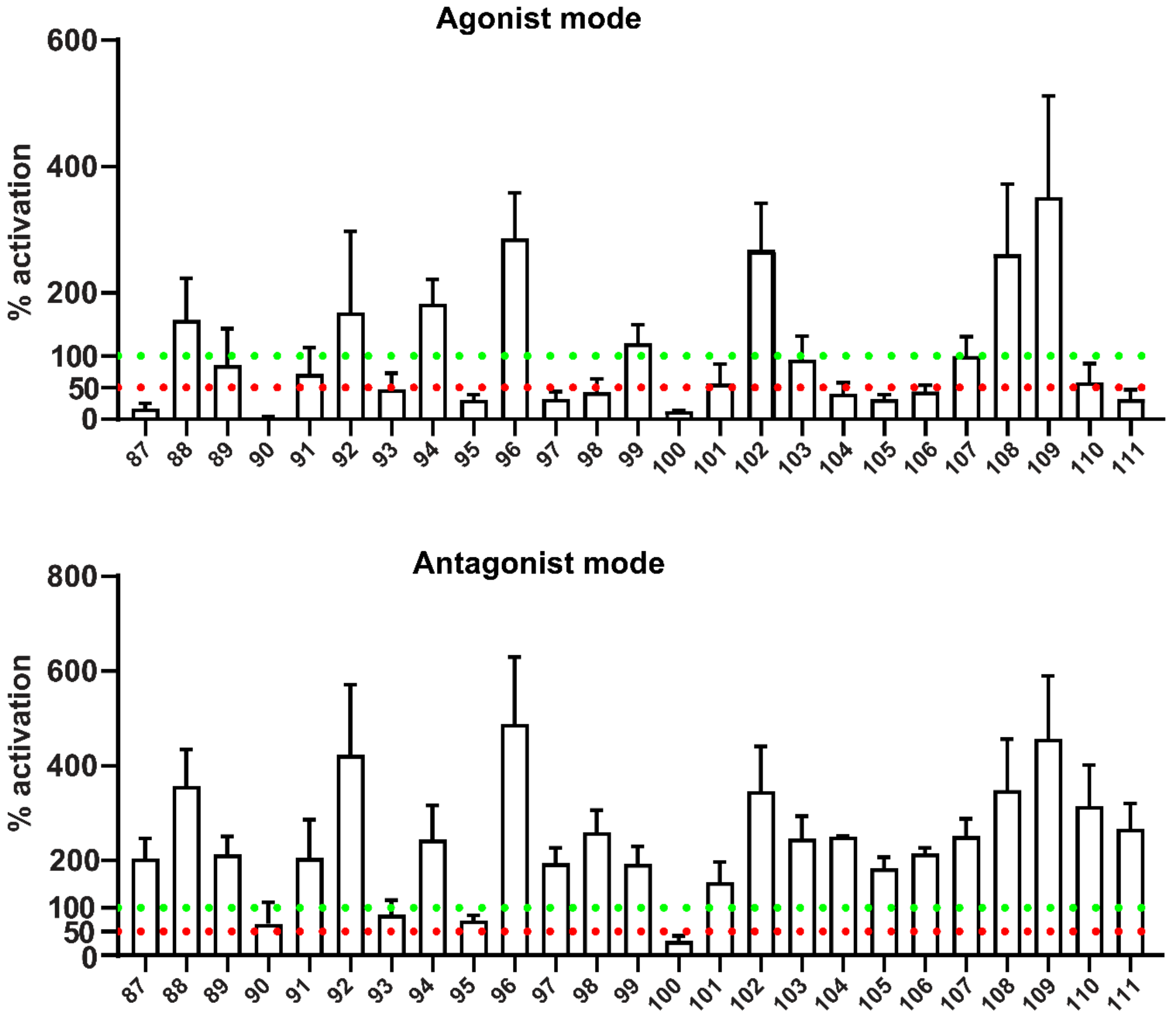

Supplementary Figure S3. Effects of second round structural analogues (A) alone or (B) in combination with rifampicin on PXR-mediated transactivation of CYP3A4 reporter gene. H-P cells were transiently transfected with CYP3A4 reporter gene, treated with 0.1% (A) or 0.2% (B) DMSO, 10 μM rifampicin, 10 μM of test compounds or co-treated with 10 μM rifampicin and 10 μM test compounds. Luciferase activities were measured after 24 h treatment. Data is expressed as mean ± SD %. Activation was calculated according to Zhu et al. [38] from three independent experiments with technical triplicates. Fold induction achieved by 10 μM rifampicin was set as 100%. Green and red dashed lines represent the 100% and 50% activation, respectively.

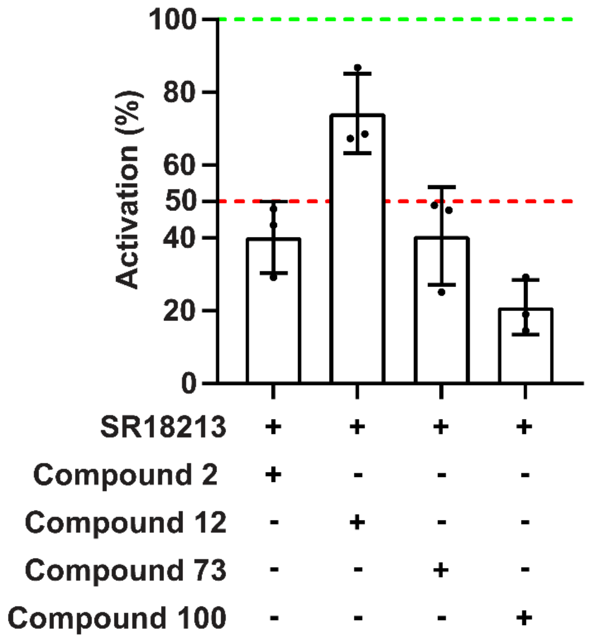

Supplementary Figure S4. Effects of potential novel PXR antagonists in combination with 1 µM SR18213 on PXR-mediated transactivation of CYP3A4 reporter gene. H-P cells were transiently transfected with CYP3A4 reporter gene, treated with 0.2% DMSO, and co-treated with 1 µM SR18123 and 10 µM test compounds. Luciferase activities were measured after 24 h treatment. Data is expressed as mean ± SD %. Activation was calculated according to Zhu et al. [38] from three independent experiments with technical triplicates individual experiments illustrated with dots. Fold induction achieved by 1 µM SR12813 was set as 100%. Green and red dashed lines represent the 100% and 50% activation, respectively.

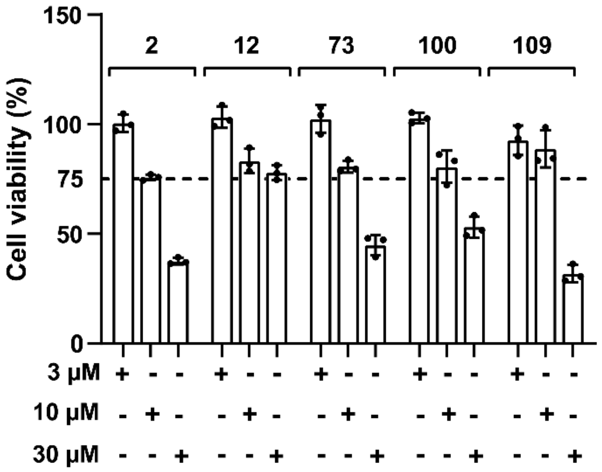

Supplementary Figure S5. Cell viability of HepG2 cells following 24 h treatment with potential novel PXR ligands. HepG2 cells were seeded, and on following day treated with 0.1% DMSO, 3, 10 and 30 µM of test compounds. Cell viabilities were measured after 24 h incubation with treatments using CellTiter-Glo assay. Cell viability in the presence of vehicle DMSO only was set as 100%. Results are expressed as mean ±SD from three independent experiments with technical triplicates and individual experiments illustrated with dots.

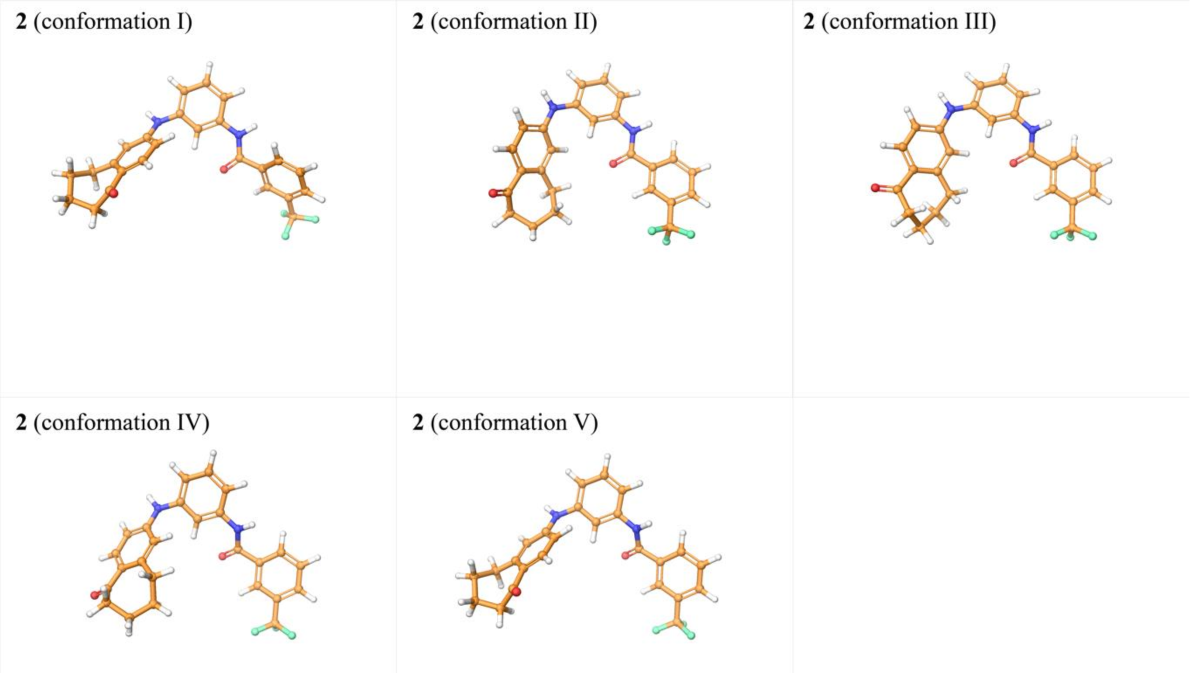

Supplementary Figure S6. QM Conformer & Tautomer Predictor output conformations of compound **2**.

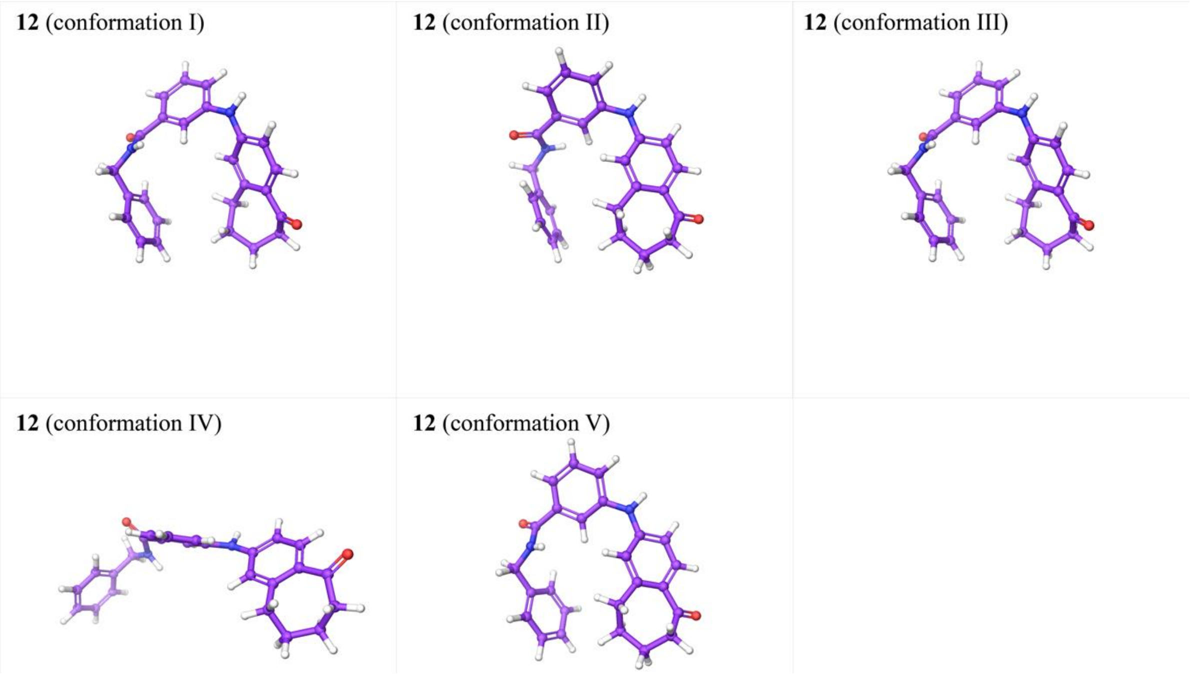

Supplementary Figure S7. QM Conformer & Tautomer Predictor output conformations of compound **12**.

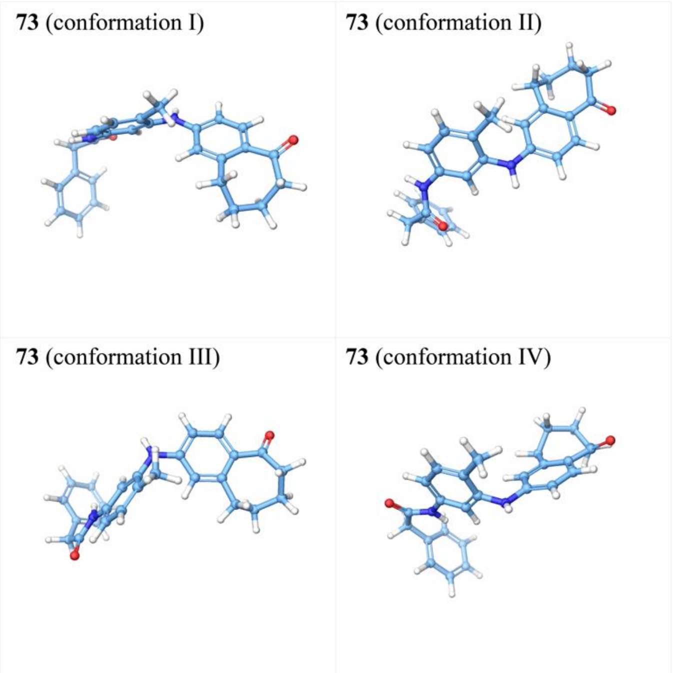

Supplementary Figure S8. QM Conformer & Tautomer Predictor output conformations of compound **73**.

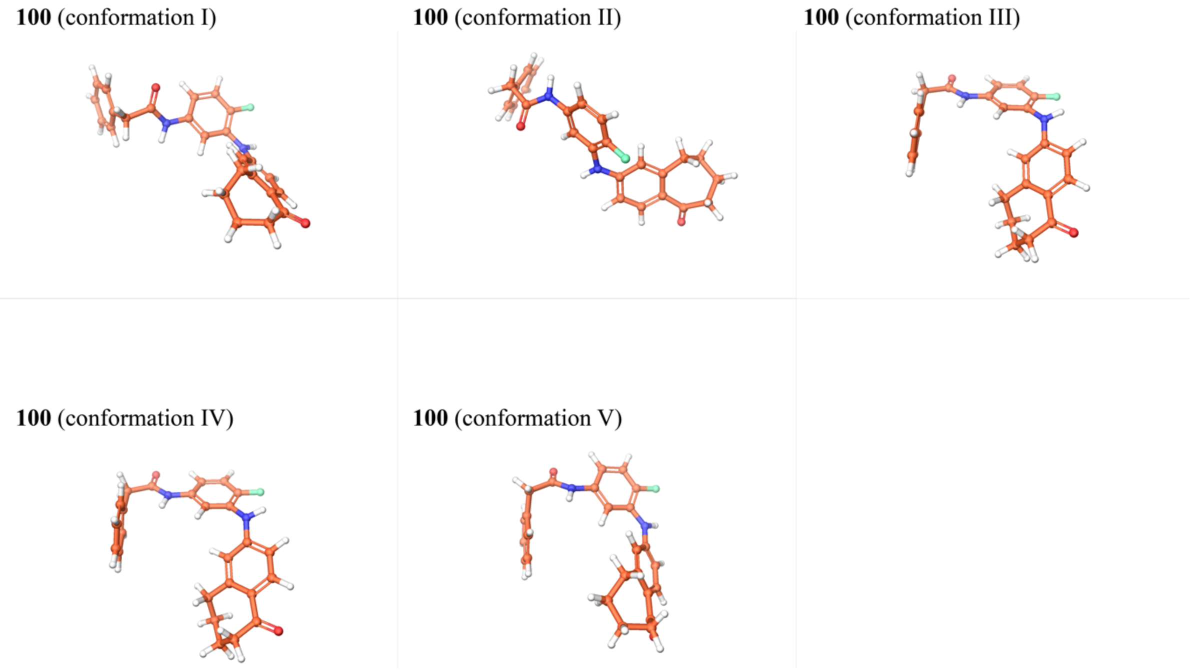

Supplementary Figure S9. QM Conformer & Tautomer Predictor output conformations of compound **100**.

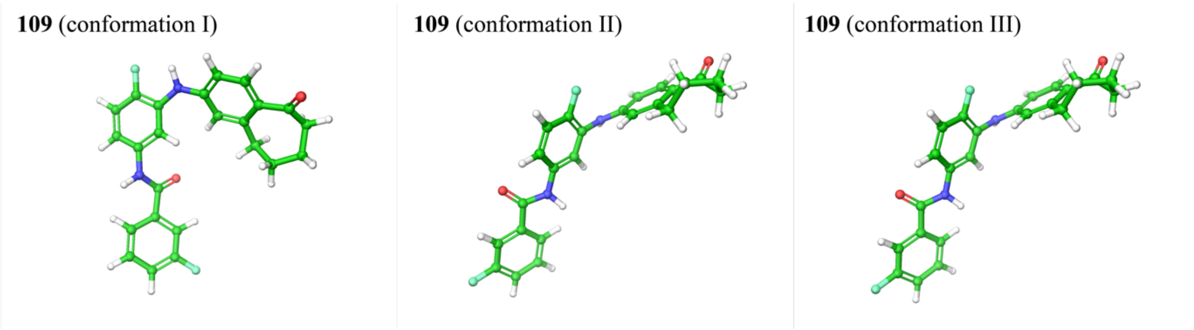

Supplementary Figure S10. QM Conformer & Tautomer Predictor output conformations of compound 109.

Supplementary Tables

Supplementary Table S1. QM Conformer & Tautomer Predictor output conformations and their energies. See SI Figures S6–S10 for the structures.

Compound	Solution phase energy	Boltzmann Population	Conformation extended/folded
**2** (conformation I)	-1524.787992	63.152	(Extended[a])
**2** (conformation II)	-1524.786778	17.450	(Folded[a])
**2** (conformation III)	-1524.786163	9.093	(Folded[a])
**2** (conformation IV)	-1524.785900	6.886	(Folded[a])
**2** (conformation V)	-1524.785239	3.419	(Extended[a])
**12** (conformation I)	-1227.019389	27.161	Folded
**12** (conformation II)	-1227.019368	26.585	Folded
**12** (conformation III)	-1227.019349	26.040	Folded
**12** (conformation IV)	-1227.018569	11.406	Extended
**12** (conformation V)	-1227.018325	8.808	Folded
**73** (conformation I)	-1266.331113	46.333	Extended
**73** (conformation II)	-1266.330934	38.341	Extended
**73** (conformation III)	-1266.329904	12.872	Extended
**73** (conformation IV)	-1266.328339	2.454	Extended
**100** (conformation I)	-1326.266051	61.882	Extended
**100** (conformation II)	-1326.265065	21.787	Extended
**100** (conformation III)	-1326.264220	8.896	Folded
**100** (conformation IV)	-1326.263458	3.970	Folded
**100** (conformation V)	-1326.263329	3.466	Folded
**109** (conformation I)	-1386.203087	38.610	(Folded[a])
**109** (conformation II)	-1386.202939	32.988	(Extended [a])
**109** (conformation III)	-1386.202797	28.402	(Extended [a])

[a]Compounds **2** and **109** have shorter R_2-group which renders their conformational space different from the other compounds.

Supplementary Table S2. PXR activating protein kinase inhibitors.

Protein kinase inhibitor	Reference
Brigatinib	(FDA, 2017) = ref. [65]
Dabrafenib	(Creusot et al., 2020) = ref. [15]
Encorafenib	(FDA, 2018) = ref. [63]
Erlotinib	(Harmsen et al., 2013) = ref. [13]
Gefinitib	(Harmsen et al., 2013) = ref. [13]
Lorlatinib	(FDA, 2018) = ref. [64]
Nilotinib	(Harmsen et al., 2013) = ref. [13]
Sorafenib	(Harmsen et al., 2013) = ref. [13]
Vandetanib	(Harmsen et al., 2013) = ref. [13]
Vemurafenib	(MacLeod et al., 2015) = ref. [67]
